# Neuroglial Senescence, α-Synucleinopathy, and the Therapeutic Potential of Senolytics in Parkinson’s Disease

**DOI:** 10.3389/fnins.2022.824191

**Published:** 2022-04-19

**Authors:** Sean J. Miller, Cameron E. Campbell, Helen A. Jimenez-Corea, Guan-Hui Wu, Robert Logan

**Affiliations:** ^1^Pluripotent Diagnostics Corp. (PDx), Molecular Medicine Research Institute, Sunnyvale, CA, United States; ^2^Department of Biology, Eastern Nazarene College, Quincy, MA, United States; ^3^Department of Neurology, Suzhou Municipal Hospital, The Affiliated Suzhou Hospital of Nanjing Medical University, Suzhou, China

**Keywords:** Parkinson’s disease, senescence, α-synuclein, astrocyte, microglia

## Abstract

Parkinson’s disease (PD) is the most common movement disorder and the second most prevalent neurodegenerative disease after Alzheimer’s disease. Despite decades of research, there is still no cure for PD and the complicated intricacies of the pathology are still being worked out. Much of the research on PD has focused on neurons, since the disease is characterized by neurodegeneration. However, neuroglia has become recognized as key players in the health and disease of the central nervous system. This review provides a current perspective on the interactive roles that α-synuclein and neuroglial senescence have in PD. The self-amplifying and cyclical nature of oxidative stress, neuroinflammation, α-synucleinopathy, neuroglial senescence, neuroglial chronic activation and neurodegeneration will be discussed. Finally, the compelling role that senolytics could play as a therapeutic avenue for PD is explored and encouraged.

## Introduction

Parkinson’s disease (PD) is the most common neurodegenerative disease that is primarily associated with the loss of motor function. It is also the second most prevalent neurodegenerative disease besides Alzheimer’s disease. Although PD is considered primarily a movement disorder, it can present with severe non-motor symptoms such as impaired bladder control, sleep disturbances, emotional disturbances, and constipation. The risk for PD increases with age, male gender, pesticide exposure, and melanoma (Chen et al., 2017; Delamarre and Meissner, 2017; Ye et al., 2020). Conversely, PD risk has an inverse relationship with nicotine use, caffeine intake, and urate levels (Chen et al., 2013; Bakshi et al., 2015; Delamarre and Meissner, 2017; Marras et al., 2019). Several gene mutations have been associated with increased risk. Familial PD is linked to such genes as SNCA, PRKN, LRRK2, PINK1, FBX07, PLA2G6, and others (Blauwendraat et al., 2020). Sporadic PD cases have been associated with genetic mutations in genes such as GBA, ACMSD, STK39, NMD3, STBD1, GPNMB, FGF20, MMP16, STX1B, ITGA8, and others (Chai and Lim, 2013). The underlying pathophysiology of PD is linked to oxidative stress and inflammation (Hald and Lotharius, 2005; Chen et al., 2018). Recent and compelling discussions have also highlighted the role of lipidopathy in PD pathology (Fanning et al., 2020). However, proteinopathy is the pathological hallmark of the disease, as it is in many neurodegenerative diseases.

The primary focus of neurodegenerative pathophysiology has been historically centered on the role of misfolded pathogenetic proteins. For example, amyloid-beta peptides are implicated in Alzheimer’s disease, TAR DNA-binding protein 43 is implicated in amyotrophic lateral sclerosis and frontotemporal lobar degeneration, the huntingtin protein is implicated in Huntington’s disease, and α-synuclein is implicated in PD (Arrasate and Finkbeiner, 2012; Halliday et al., 2012; Stefanis, 2012; Blokhuis et al., 2013; Cheignon et al., 2018). Furthermore, many neurodegenerative diseases involve aggregation of the tau protein, including Alzheimer’s disease, amyotrophic lateral sclerosis, frontotemporal lobar degeneration, and PD (Spillantini and Goedert, 2013; Eftekharzadeh et al., 2018).

Aging is the greatest risk factor for developing PD (Reeve et al., 2014). The nine classic hallmarks of cellular aging include genomic instability, telomere attrition, epigenetic alterations, loss of proteostasis, deregulated nutrient sensing, mitochondrial dysfunction, cellular senescence, stem cell exhaustion, and finally, altered intercellular communication, which is linked to chronic inflammation (López-Otín et al., 2013). The Geroscience Hypothesis identifies the following seven “pillars of aging”: macromolecular damage, epigenetics, inflammation, adaptation to stress, proteostasis, stem cells and regeneration, and metabolism (Kennedy et al., 2014).

Senescent cells are intrinsically linked to the aging process. The senescence-associated secretory phenotype (SASP) of senescent cells releases pro-inflammatory cytokines, chemokines, and promotes inflammation (Coppé et al., 2008). The recently developed Unitary Theory of Fundamental Aging Mechanisms describes the cellular facets of aging as being so closely interrelated that therapeutic targeting of one aspect, such as cellular senescence, might mitigate many, or all, of the others (Tchkonia et al., 2021). Furthermore, the Unitary Theory of Fundamental Aging Mechanisms identifies several other additional aging-related hallmarks such as increased fibrosis, increased CD38, decreased NAD^+,^ and the accumulation of misfolded and aggregated proteins (Tchkonia et al., 2021).

Operating under the Unitary Theory of Fundamental Aging Mechanisms, a direct relationship should exist between senescent cells and proteinopathy. Since most of the research conducted on senescent cells has focused on peripheral tissues, it is especially of interest to explore the relationship between senescent cells and proteinopathy in the central nervous system (CNS) (Baker and Petersen, 2018). Here, we explore the relationship between α-synucleinopathy, senescent astrocytes, and senescent microglia in PD. Additionally, the potential of senolytics for therapy in PD will be discussed.

## α-Synucleinopathy in Parkinson’s Disease

The protein α-synuclein is small (14 kDa), soluble, intrinsically unstructured, and encoded by the *SNCA* gene (Uversky, 2003). The intrinsically disordered nature of monomeric α-synuclein is stable and conserved across mammalian cell types (Theillet et al., 2016). α-synuclein is located ubiquitously in CNS presynaptic terminals (Jakes et al., 1994). Although the normal function of α-synuclein is not well understood, it is known to be involved in some regulatory roles such as neurotransmitter release and synaptic plasticity, dopamine metabolism, membrane remodeling, and DNA repair (Bendor et al., 2013; McCann et al., 2014; Schaser et al., 2019). The most common α-synuclein isoform found in humans is 140 amino acids long (Jakes et al., 1994; Goedert et al., 2017).

**FIGURE 1:**
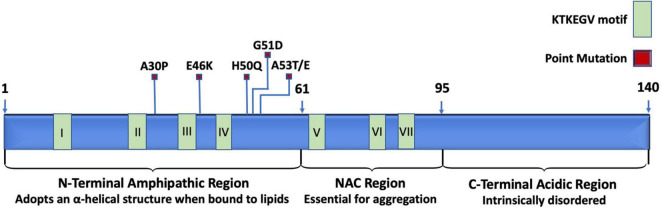
Schematic representation of relevant landmarks on the protein α-synuclein. The N-terminus is characterized by being amphipathic, containing mutations associated with familial PD, four of the seven KTKEGV repeats, and lipid-binding sites that induce the disordered structure to adopt an α-helical secondary structure when bound to lipid membranes. The central non-amyloid-β component (NAC) is hydrophobic and is the site where α-synuclein proteins interact with each other to form aggregates. The C-terminus is acidic and intrinsically disordered.

Under normal physiological conditions, the structure of α-synuclein resists aggregation. The N-terminal region is amphipathic, has a basic pH, binds to membranes, and changes from a disordered structure to an α-helical structure when bound to lipids (Bartels et al., 2010, 2011; Theillet et al., 2016). The N-terminal region spans the first 60 residues of α-synuclein and is the location of three familial PD mutations: A30P, E46K, and A53T (Ono, 2017). The N-terminal region also includes the beginning of a stretch of seven imperfect repeats of “KTEKEGV” (Dettmer et al., 2015). N-terminal acetylation destabilizes α-synuclein, increases α-synuclein levels, and enhances α-synuclein toxicity (Vinueza-Gavilanes et al., 2020). The central core spans from residues 61 to 95 and consists of hydrophobic amino acids. The central region is also referred to as the non-amyloid-β component (NAC) and is the site essential for misfolding and aggregation (Ono, 2017). In wild-type α-synuclein, the NAC is protected from cytoplasmic exposure due to long-range interactions between the N-terminal and the C-terminal, which acts to prevent aggregation (Bertoncini et al., 2005; Theillet et al., 2016). Additionally, chaperones are known to bind to the N-terminus around tyr39, which further helps to prevent aggregation (Burmann et al., 2020). Mutations in the N-terminal have been shown to disrupt the interaction between the N-terminal and the C-terminal to promote the pathological gain-of-function α-synuclein aggregation (Bertoncini et al., 2005). The remainder of the imperfect “KTEKEGV” repeated motifs are found in the NAD region. The C-terminal is intrinsically disordered and is highly acidic (Suzuki et al., 2018). The structure of α-synuclein is depicted in Figure 1.

**FIGURE 2:**
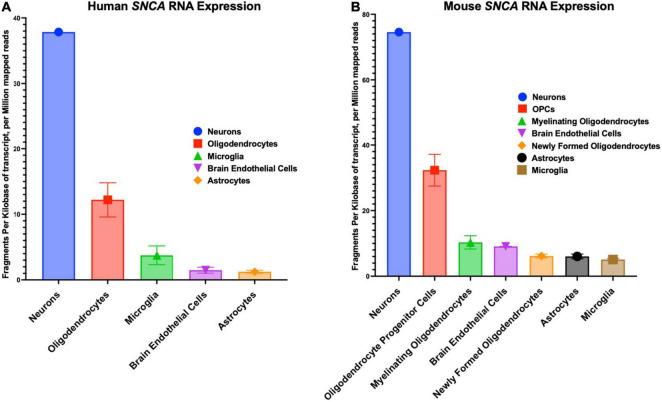
Cellular distribution in the central nervous system of *SNCA* RNA expression. **(A)** Human cellular distribution of SNCA transcripts. **(B)** Mus musculus cellular distribution of SNCA transcripts. RNA-Seq data used for these graphs were obtained and adapted from the Ben Barres lab’s brain RNA-seq database, www.brainrnaseq.org. The original mouse data came from Zhang et al. (2014) and the original human data came from Zhang et al. (2016).

The *SNCA* gene in humans has a chromosomal location of 4q22.1, a length of 114,226 base pairs, and contains six exons (Touchman et al., 2001). *SNCA* transcription is regulated by beta-2-adrenoreceptor (B2AR), which can be antagonized to increase the risk of PD or activated to reduce the risk of PD (Mittal et al., 2017). The cellular distribution patterns of various SNCA transcript quantities among humans and mice are shown in Figure 2. The first genetic mutation identified to be associated with PD is the G-to-A transition at the 209*^th^* nucleotide, which results in the A53T mutation of the *SNCA* gene located between the 4*^th^* and 5*^th^* repeat of KTEKEGV (Goedert, 1997; Polymeropoulos et al., 1997; Stefanis, 2012). The familial and highly penetrant A53T mutation is inherited in an autosomal dominant manner and is associated with early onset PD (Puschmann et al., 2009). In rat dopaminergic PC12 cells, the A53T mutation has been shown to induce α-synuclein related cell death due to reduced proteasome activity, increased reactive oxygen species (ROS), increased mitochondrial permeability and dysfunction, cytochrome C release, increased activity of caspase-3, caspase-9, and caspase-12, and finally, endoplasmic reticulum (ER) stress due to α-synuclein accumulation in the ER (Tanaka et al., 2001; Smith et al., 2005; Colla, 2019).

Sequence mutations in the *SNCA* gene, such as A53T, are known to increase the rate and magnitude of α-synuclein aggregation. Subsequent to intracell α-synuclein aggregation, dopamine accumulates in the cytoplasm and dopaminergic toxicity increases in severity (Tabrizi et al., 2000). Duplications or triplications of wild-type *SNCA* is also implicated in PD pathology, PD with dementia, dementia with Lewy bodies and multiple system atrophy (Book et al., 2018). *SNCA* triplication is linked to early onset autosomal dominant familial PD and PD-related dementia, as demonstrated in the Spellman-Muenter kindred, a Swedish-American family, a family from Italy, and several others (Singleton et al., 2003; Farrer et al., 2004; Olgiati et al., 2015; Zafar et al., 2018). Human carriers of the triplicate mutation of *SNCA* had twice the control levels of α-synuclein mRNA in blood and brain tissue (Miller et al., 2004). Soluble α-synuclein protein levels were also doubled in the blood of the triplicate *SNCA* carriers, whereas the genomic triplication of *SNCA* led to greater levels of insoluble α-synuclein aggregates in the brain (Miller et al., 2004). DA neurons differentiated from human-induced pluripotent stem cells (hiPSCs) from a *PARK4* patient who also had *SNCA* triplication showed increased levels of α-synuclein compared to control hiPSC-derived DA neurons (Fukusumi et al., 2021). Genomic duplication of *SNCA* also increases α-synuclein levels and is causal for familial PD (Chartier-Harlin et al., 2004; Ibáñez et al., 2004). There is well established direct relationship between *SNCA* copy number, α-synuclein abundance, and PD phenotype severity (Singleton and Gwinn-Hardy, 2004). In contrast to triplication, cases of *SNCA* duplication resemble idiopathic PD with a late onset, slow progression, and are spared from dementia (Chartier-Harlin et al., 2004; Ibáñez et al., 2004). However, there has been a single case described recently of a male with *SNCA* duplication who developed early onset PD with aggressively progression and rapid cognitive decline (Kielb et al., 2021).

The abnormal accumulation of phosphorylated α-synuclein into insoluble aggregate is characteristic of Lewy bodies and Lewy neurites and is the defining histopathological hallmark of α-synucleinopathies. The three main α-synucleinopathy diseases include PD, Lewy Body Dementia (LBD), and multiple system atrophy (MSA) (McCann et al., 2014). The most common α-synucleinopathy disease is PD (Grazia and Goedert, 2000). Lewy neurites mostly have a course, thin and elongated appearance (Braak et al., 1999). They are located in the cytoplasm and are greater in number than Lewy bodies, especially in the striatum and amygdala (Volpicelli-Daley et al., 2014). They are also heavily distributed in the dorsal vagal nucleus, the CA2/3 hippocampus region, and the nucleus basalis of Meynert (Kon et al., 2020). Lewy neurites have been shown to impair axonal transport of autophagosomes and endosomes that contain Rab7 and TrkB receptors (Volpicelli-Daley et al., 2014).

Lewy bodies are well-defined, spherical protein conglomerates composed of misfolded α-synuclein and other components. They are present in PD patients except for a handful of unique familial cases (Johansen et al., 2018). Lewy bodies are located in the neuronal cytoplasm and are found distributed across the brain stem, limbic areas, and neocortical brain regions (Rezaie et al., 1996; Spillantini and Goedert, 2000). Lewy body accumulation correlates aging, the severity of PD, and severity of dementia (Saito et al., 2004). Likewise, α-synuclein in Lewy bodies is phosphorylated and nitrated, indicating oxidative stress is intrinsic to their formation (Giasson et al., 2000; Foulds et al., 2011; McCormack et al., 2012; Wang et al., 2012; Kellie et al., 2014). Although widely recognized as contributing to neurodegeneration, there is some debate over whether Lewy bodies serve a protective role in the cell, if the process of forming the Lewy body promotes neurodegeneration, or if the Lewy body itself promotes neurodegeneration (Ono, 2017; Iqbal et al., 2020; Mahul-Mellier et al., 2020). All three of these hypotheses are likely partially true. Furthermore, Lewy body composition has received renewed attention recently, where the role of both α-synuclein and non- α-synuclein components are being reassessed. Some non- α-synuclein components of interest include ubiquitin, damaged organelles such as fragmented mitochondria, and lipids (Nakamura et al., 2011; Lashuel, 2020). It is important to note that there is evidence to suggest that α-synuclein is not the most abundant constituent of Lewy bodies, contrary to the filament-centric dogma (Lashuel, 2020).

Misfolded proteins in neurodegenerative diseases have been shown to exist both intracellularly and extracellularly (Peng et al., 2020). In PD, α-synuclein aggregations are seen earliest in the disease progression to be located in the olfactory bulb and the dorsal motor nucleus of the tenth cranial nerve (Peng et al., 2020; Wakabayashi, 2020). The pathological α-synuclein then spreads rostrally through the brainstem, midbrain, forebrain, and eventually to the cortex (Braak and Del Tredici, 2017). α-synuclein has been shown to pass between neurons, from neurons to microglia, from neurons to astrocytes, between astrocytes, and across the blood-brain barrier (BBB) (Fellner et al., 2013; Loria et al., 2017; Rostami et al., 2017; Bogale et al., 2021).

### Microglial α-Synucleinopathy in Parkinson’s Disease

Microglia are the CNS’s resident immune macrophage that monitors for homeostatic threats and intervenes when necessary. Along with other glial populations, microglia are highly diverse based on their neuroanatomical location and functional plasticity, suggesting that they are influenced by local environment cues (Olah et al., 2011; Bachiller et al., 2018; Li and Barres, 2018; Kam et al., 2020). For example, microglia in healthy mouse basal ganglia regions had region-specific morphology, cell density, and count, lysosome content and distribution, membrane resting potentials, and transcriptomes (De Biase et al., 2017; Costa et al., 2021). Additionally, microglia experience altered intracellular α-synuclein levels based on their environment, such as in response to cytokines or cerebrospinal fluid (CSF) from PD patients (Bick et al., 2008; Schiess et al., 2010).

Substantia nigra pars compacta (SNpc) microglia differ from microglia in the ventral tegmental area (VTA) (Shaerzadeh et al., 2020). Perhaps regional microglial differences might partly explain the PD-related loss of dopaminergic neurons in the SNpc, rather than in the VTA. The reasons for this regional selectivity of dopaminergic neuronal loss are not yet fully understood (Krashia et al., 2019; Shaerzadeh et al., 2020). However, it seems like a reasonable hypothesis that microglial activation has a role to play. For example, mice overexpressing wild-type human α-synuclein throughout the CNS had increased levels of activated microglia and TNF-alpha in the striatum as early as 1 month old and then the substantia nigra as early as 5 months old, but not in other brain areas (Watson et al., 2012). The region-specific activated microglial response to increased levels of α-synuclein persisted as long as monitoring took place, over 14 months (Watson et al., 2012). In human PD patients, PET imaging and postmortem brain analysis showed regionally activated microglial cells in the midbrain, the frontal cortex, and the temporal cortexes (Gerhard et al., 2006; Garcia-Esparcia et al., 2014). Both 1-Methyl-4-phenyl-1,2,3,6-tetrahydropyridine (MPTP) and α-synuclein over-expression models of PD in monkeys also showed region-specific and long-term microglial activation in the SNpc (Kanaan et al., 2008; Barkholt et al., 2012). Therefore, elevated α-synuclein levels cause microglia to become quickly and persistently activated, which leads to increased neuroinflammatory secretions from the microglia.

Among *in vitro* and *in vivo* models of PD, α-synuclein causes microglia to become rapidly activated, to migrate to the α-synuclein source, and then increases phagocytic and pro-inflammatory activity (Zhang et al., 2005; Su et al., 2008; Wang et al., 2015; Mavroeidi and Xilouri, 2021). Extracellular α-synuclein is cleared through activated microglial engulfing and autophagy, mediated by TLR4-NF-kB signaling in a process recently discovered and coined as “synucleinphagy” (Choi et al., 2020). However, microglial phagocytic activity is reduced with age (Bliederhaeuser et al., 2016). The level of microglial activation is greater in the presence of α-synuclein mutants compared to wild-type α-synuclein protein, perhaps reflecting the severity of their respective associated pathologies (Roodveldt et al., 2010; Hoenen et al., 2016). Indeed, the PD-related α-synuclein A53T mutation has been shown to increase the production of microglial CXCL12 in cell culture and in mouse SNpc (Li et al., 2019). Postmortem brain tissue of PD patients have also shown a direct correlation between α-synuclein and CXCL12 levels (Li et al., 2019).

### Astrocytic α-Synucleinopathy in Parkinson’s Disease

Astrocytes are the most abundant cell type in the CNS, constituting an estimated 20–40% of the mammalian brain and are about five times as prevalent as neurons (Khakh and Sofroniew, 2015; Troncoso-Escudero et al., 2018; Giovannoni and Quintana, 2020). Over twenty different structural and functional subpopulations of astrocytes exist in the adult human CNS (Miller, 2018; Verkhratsky and Nedergaard, 2018). Despite their heterogeneity, the overarching function of astrocytes is to provide support for neurons and to help ensure a homeostatic environment in the brain. Toward this end, astrocytes perform many functions. For example, they contribute to BBB maintenance, promote normal synaptic function, secrete neurotrophic molecules, modulate their microenvironment as necessary, and are involved with the regulation of neurogenesis, lipoprotein secretion, and cerebral blood flow (Ding et al., 2021). Additionally, in experimental mouse models with either ablated or dysfunctional microglia, astrocytes became activated and phagocytic to help compensate for the microglial loss (Konishi et al., 2020).

Even in their reactive state, astrocytes exhibit functional variability. From rest (A0), astrocytes can enter one of two polarized forms of activation based on their gene expression profile in response to stimuli: A1 or A2. In broad terms, A1 is neurotoxic and A2 is neuroprotective (Zamanian et al., 2012; Liddelow and Barres, 2017; Ding et al., 2021). Astrocytes and microglia share a close relationship regarding reactivity and phagocytosis. Simply stated, reactive microglia give rise to astrocyte reactivity (Liddelow et al., 2017). Indeed, the cytokines Il-1α, TNF-α, and C1q, which are released from reactive M1 microglia during chronic neurodegenerative conditions, are both necessary and sufficient for producing an A1 phenotype in astrocytes (Liddelow et al., 2017). Blocking the ability of microglia to activate A1 astrocytes helps to preserve *in vivo* dopaminergic neuron viability (Hinkle et al., 2019). A1 reactive astrocytes contribute to PD pathology by secreting a host of neurotoxic and pro-inflammatory factors that inhibit synaptogenesis and promote dopaminergic neuronal death (Liddelow and Barres, 2017). Conversely, in response to traumatic or ischemic CNS injury, M2 microglia induce the genetic upregulation and downregulation of astrocytes to exert a neuroprotective A2 phenotype (Hernández et al., 2021). A2 astrocytes become reactive in response to microglial secreted IL-1β, IL-6, and nuclear factor IA (NFIA) as well as the silencing of miR-21 (Su et al., 2019; Tchieu et al., 2019).

The A1 astrocyte phenotype has been observed in cultured astrocytes treated with α-synuclein aggregates (Lee et al., 2010; Troncoso-Escudero et al., 2018). Furthermore, cultured astrocytes readily take up secreted α-synuclein aggregates directly from neighboring co-cultured neurons to adopt an A1 phenotype (Lee et al., 2010). Neuronally secreted α-synuclein binds to the toll-like receptors (TLRs) of astrocytes and promotes an increased neuroinflammatory response through activated TLR signaling (Lee et al., 2010; Fellner et al., 2013; Rannikko et al., 2015; Booth et al., 2017; Verkhratsky and Nedergaard, 2018). The direct relationship between α-synuclein aggregates and reactive A1 astrocytes have also been observed *in vivo*, through a transgenic mouse model that expressed human α-synuclein (Lee et al., 2010). Additionally, in postmortem PD patient brains, inclusions of α-synuclein aggregates have been found in astrocytes as well as in neurons (Wakabayashi et al., 2000; Hishikawa et al., 2001; Braak et al., 2007; Booth et al., 2017). When mutated α-synuclein is selectively expressed in mouse astrocytes, the astrocytes develop the A1 phenotype, neuroinflammation increases, microglia become activated and mice develop paralysis (Gu et al., 2010). Despite the detrimental effects of reactive astrocytes induced by α-synuclein, astrocytes also contribute to the degradation and removal of mutated α-synuclein and are more efficient at it than neurons are (Tsunemi et al., 2020).

## Brain Cellular Senescence in Parkinson’s Disease

### Introduction to Cellular Senescence

Three fates ultimately await very aged or damaged cells: senescence, apoptosis, or autophagy (Vicencio et al., 2008). Autophagy (self-eating) is largely a homeostatic mechanism through lysosomal destruction of old or damaged cellular components, allowing for the recycling of cellular material. However, recent evidence also supports the view that autophagy plays an important role in mammalian cell death (Jung et al., 2020). Apoptosis (self-killing) is a morphologically unique, genetically programmed cell death. It has important roles in development, aging, and atrophy, to maintain an appropriate level of cells (Elmore, 2007). It also has many critical roles in immune system function (Nagata and Tanaka, 2017). Cells can also enter a state of senescence (stable cell cycle arrest) as a homeostatic response to various stressors in order to mitigate the proliferation of damaged cells and to prevent neoplastic transformation (Kritsilis et al., 2018). Senescent cells are metabolically active, stable, and viable, unlike cells destined for death and recycling. The classical understanding is that only mitotically active cells in the periphery can enter senescence by becoming arrested in the G_1_ phase, unlike quiescent cells that arrest in G_0_ (Di Leonardo et al., 1994; Vicencio et al., 2008). However, post-mitotic cells in the central nervous system are now recognized as being able to undergo senescence (Baker and Petersen, 2018). There is some evidence of crosstalk and shared regulated pathways between these three cell fate states, but there is much that is currently not understood about their relationships to one another (Abate et al., 2020). Also, there is some debate currently over the post-mitotic status of glia and CNS neurons.

Senescent cells accumulate during aging, both from an increased rate of production and a decreased clearance rate (Karin and Alon, 2021). Although the accumulation of senescent cells is associated with increasing age, it is not age-dependent. Senescence is a dynamic and context-specific state that involves many biochemical pathways, such as the p53/p21^*WAF*1/CIP1^ and p16^*INK*4*A*^/pRB tumor suppression pathways (McConnell et al., 1998; Kumari and Jat, 2021). Senescent cells that form in response to stress during earlier life stages, such as from oncogene activation, inflammation, or DNA damage, contribute to protective functions such as tumor suppression, wound healing, preventing the propagation of tissue damage, and embryogenesis (Storer and Keyes, 2014; Ovadya and Krizhanovsky, 2018). In non-pathological states, senescent cells attract the immune system and are cleared (Langhi Prata et al., 2018).

There is a physiological threshold where senescent cells interfere with their own clearance, described by the recently proposed “immune threshold theory of senescent cell burden” (Tchkonia et al., 2021). According to this theory, once the saturation of senescent cells passes the senescent cell abundance threshold, the spread of cellular senescence outpaces the immune system’s ability to clear them and cellular senescence becomes “self-amplifying” and contributes to age-associated diseases (Tchkonia et al., 2021). The age-associated nature of senescent cell accumulation and the threshold theory of senescent cell burden makes it a clear example of evolutionary antagonistic pleiotropy (Mitteldorf, 2019).

Senescent cells have characteristic phenotypes marked by irreversible and permanent cell cycle arrest, SASP, disruption of normal mitochondrial form and function, metabolic changes, genomic DNA damage, telomere attrition, altered epigenetics, impaired DNA repair mechanisms, altered proteostasis, increased reactive oxygen species (ROS) production and increased ROS-mediated damage (Martínez-Cué and Rueda, 2020). The phenotypical changes that senescent cells undergo allow the cell to influence its immediate environment through SASP as well as to ensure cell cycle arrest (Birch and Gil, 2020). The hypersecretory phenotype SASP is a key hallmark of senescent cells. The composition of SASP includes pro-inflammatory cytokines such as IL-6 and IL-8 as well as insulin-like growth factors IGFBP3, IGFBP4, and IGFB7, which have pro-senescence and tumor-suppressant properties (Özcan et al., 2016; Soto-Gamez and Demaria, 2017). In addition to cytokines and growth factors, SASP also includes chemokines, matrix-metalloproteinases, and many other constitutes (Cuollo et al., 2020). The exact composition of SASP is dynamic and heterogeneous depending on the senescent cell type, driver of senescence, and cellular context (Coppé et al., 2011; Maciel-Barón et al., 2016; Birch and Gil, 2020).

Senescence-associated secretory phenotype works to reinforce the cellular senescence of its source through the autocrine senescence process as well as spreading senescence to non-senescent neighboring cells *via* paracrine senescence (Hoare and Narita, 2013; Borodkina et al., 2018). SASP can be activated in various ways, but it has been argued that DNA damage is the main upstream driver (Kumari and Jat, 2021). The NF-κB and JAK2/STAT1 pathways are the main regulators of SASP (Gao et al., 2021). Therefore, SASP expression is influenced by pathways that modulate the NF-κB and JAK2/STAT1 pathways, such as the mammalian target of rapamycin (mTOR) pathway, mitogen-activated protein kinase (MAPK) signaling, the phosphoinositide 3 kinase (PI3K) pathway, the DNA damage response (DDR), and GATA4/p62-mediated autophagy (Soto-Gamez and Demaria, 2017; Birch and Gil, 2020).

### Microglial Senescence in Parkinson’s Disease

Senescent microglia differ from activated microglia. Microglia undergo non-pathologic changes related to advanced age that brings them to a senescent state, sometimes synonymously referred to as dystrophy, although dystrophy typically only refers to the morphological changes of senescent microglia (Streit et al., 2004, 2009, 2014; Angelova and Brown, 2019; Shahidehpour et al., 2021). Dramatic morphological dystrophy of senescent microglia is not seen in the brains of mice but is observed in humans (Streit et al., 2014). Senescent microglia undergo distinct age-related morphological, functional, and distribution changes (Streit et al., 2014; Shaerzadeh et al., 2020; Brawek et al., 2021). For example, senescent microglia experience swelled spherical somas, a fragmented and beaded cytoplasm, and reduced ramification (very shortened processes, a reduction in the number of processes, and a reduction in the number of branches per process) (Shaerzadeh et al., 2020; Brawek et al., 2021). Senescent microglia also experience a change in their uniform distribution resulting in increased location-specific density (Sikora et al., 2021). The total number also increases with age, although this seems debatable (Kodama and Gan, 2019; Ritzel et al., 2019; Sikora et al., 2021).

Functionally, senescent microglia resemble a mild activation state (Lopes et al., 2008; Streit et al., 2014; Rodriguez et al., 2015). For example, there is an increased proportion of senescent microglia in the aged brain that experience spontaneous Ca^2+^ transients compared to middle-aged or young brains in mice (Del Moral et al., 2019). Neurodegenerative insult still elicits an attempt by aged microglia to boost their heightened baseline activation levels. For example, aged mouse models of Alzheimer’s disease experience a higher degree of intracellular Ca^2+^ transients in senescent microglia than what is seen due to aging alone (Brawek et al., 2014). Additionally, senescent microglia have a reduced rate of migration to injured areas and a reduced rate of process movement toward injury (Wong, 2013; Brawek et al., 2021). Furthermore, aged mice have impaired microglial phagocytic ability (Ritzel et al., 2019). As the proportion of senescent microglia accumulates with age or age-related disease, they eventually outnumber healthy microglia and produce an enhanced neuroinflammatory response to injury, compared to young brains (Sugama et al., 2003; Wasserman and Schlichter, 2008; Luo et al., 2010). Together, these observations support the notion of impaired function of senescent microglia in aged brains.

As the brain ages, it experiences chronic low-level oxidative stress and subsequent inflammation (Streit et al., 2014). Aging drives the switch from neuroprotective microglia to senescent microglia, as well as an increased concentration of senescent microglia in the SNpc (Angelova and Brown, 2019). As part of the age-related inflammatory milieu of the CNS, senescent microglia chronically secrete pro-inflammatory cytokines such as IL-6, IL-8, IL-1β, and TNF-α (Sierra et al., 2007; Wong, 2013; Sikora et al., 2021). They also release a reduced amount of anti-inflammation cytokines and increased levels of ROS, which are Nox-2 dependent (Angelova and Brown, 2019; Geng et al., 2020). The pro-inflammatory secretions of senescent microglia are akin to the pro-inflammatory secretions of activated microglia. However, consistent with a senescent state, senescent microglia also have genomic and mitochondrial DNA damage as well as telomere shortening (Costa et al., 2021; Hsiao et al., 2021). Senescent microglia also have upregulated expression of senescent markers such as Bcl-2, senescence-associated β-galactosidase (SA-β-gal), p16^*INK*4*a*^, p21^*WAF*1/CIP^, lipofuscin, and H2AX[pS139] (Ritzel et al., 2019).

The senescent microglial response to injury is delayed and prolonged, which helps to shape the chronic and slowly progressive nature of PD (Luo et al., 2010; Damani et al., 2011). The significance of an aged CNS with senescent microglia in PD pathology is demonstrated clearly in mouse models. For example, a low dose of the pesticide rotenone reduced the number of SNpc dopaminergic neurons by up to 30% in old rats but was benign when given to young rats (Phinney et al., 2006). Similarly, old mice with age-related senescent microglia experienced a markedly more severe reduction of dopaminergic neuron concentration in response to MPTP injections than neonatal mice (Sawada et al., 2007). Despite the value of toxin rodent models in the study of PD, it is noted that there are some limitations in their applicability (Harms et al., 2021).

Parkinson’s disease has been epidemiologically linked to iron exposure (Angelova and Brown, 2019). Analysis of SNpc tissue from postmortem PD patients also reveal an elevated iron concentration compared to controls (Angelova and Brown, 2019). Despite not being the main cell type to store iron in the brain, senescent microglia have increased levels of ferritin, which increases their internal exposure to iron-related oxidative stress (Lopes et al., 2008; Angelova and Brown, 2019; Galaris et al., 2019). Oxidative stress in microglia, whether due to heightened iron levels or catabolizing degenerative neurons that contain iron-rich neuromelanin, prompts senescent microglial secrete pro-inflammatory factors. Therefore, in addition to aging and α-synucleinopathy, intracellular iron contributes to the senescent secretome.

There are morphological and physiological differences between male and female microglia. The sex-related differences in microglia correspond to the sex differences in PD risk. Men have between 1.5× and 2× the risk for developing PD than females (Wooten et al., 2004; Cerri et al., 2019). Furthermore, estrogens are neuroprotective against PD (Lee et al., 2019). The sex differences in microglia are thought to be the result of estrogen priming (Cerri et al., 2019). For example, there is evidence that the female 6-OHDA mouse model of PD has higher levels of estrogens than males, which prompts their activated microglia to polarize toward the neuroprotective M2 phenotype rather than the male-dominant pro-inflammatory M1 phenotype (Siani et al., 2017). The sex differences in brain inflammation response, PD risk, and microglial physiology due to estrogen activity has also been confirmed in humans (Hanamsagar et al., 2017; Villa et al., 2018; Acosta-Martínez, 2020).

### Astrocytic Senescence in Parkinson’s Disease

Senescence and reactivity are distinct astrocytic cell fates, yet they share some common features. A recent transcriptomic study revealed a wide range of senescent-related markers upregulated in senescent astrocytes such as SA-β-gal, IL6, IL8, IL1A, IL1B, *CDKN1A*, the p53/p21^*WAF*1^ and p16^*INK*4*A*^/pRB pathways, *CYR61*, *CCND1*, *IGFNP5*, and *IGFBP2* (Simmnacher et al., 2020). Senescent astrocytes also experience an upregulation of high-mobility group B (HMGB) proteins, increased production of vimentin and glial fibrillary acidic protein (GFAP), in addition to a reduced expression of neurotrophic factors and nuclear lamina protein laminB1 (Han et al., 2020). Some of the upregulated inflammatory marker genes are shared between reactive and senescent astrocytes. For example, astrocytes in both states experience upregulated pro-inflammatory cytokines, chemokines, proteases, and growth factors (Maciel-Barón et al., 2016; Cohen and Torres, 2019; Simmnacher et al., 2020). The morphology of senescent astrocytes change to become flattened, enlarged, and have vacuolized lysosomes (Bitto et al., 2010; Cohen and Torres, 2019).

Normal aging gives rise to astrocytic senescence-inducing factors such as damaged DNA and shortened telomeres (Bhat et al., 2012; Kang et al., 2015). Senescent astrocyte accumulation also occurs in neurodegenerative diseases (Limbad et al., 2020). Compared to control tissue, the SNpc tissue from five postmortem PD patients exhibited a large increase in the levels of p16^*INK*4*a*^ and the SASP components MMP-3, IL-6, IL-1α, and IL-8, as well as reduced nuclear level of lamin B1 (Freund et al., 2012; Chinta et al., 2018). Interestingly, the drop in nuclear lamin B1 levels was only observed in astrocytes, whereas nuclear lamin B1 levels remained unchanged in the astrocyte-neighboring tissues between the PD SNpc and control tissues (Chinta et al., 2018). These results suggest that astrocytes have a uniquely elevated susceptible to becoming senescent in PD. Furthermore, multiple studies have shown that cultured human and rodent astrocytes are more susceptible to toxin-induced senescence than fibroblasts (Bitto et al., 2010; Chinta et al., 2018). However, one study showed that neurons in long-term cultures of primary rat cortical cells became senescent before neuroglia did (Moreno-blas et al., 2019). It is thought that this aberrant result was due to dysfunctional age-associated autophagy (Moreno-blas et al., 2019). Senescent astrocytes also have decreased expression of glutamate transporters, and therefore, promote glutamate toxicity and subsequent death of surrounding neurons (Limbad et al., 2020).

Paraquat has been a widely used toxic herbicide and has a chemical structure similar to the dopaminergic neurotoxin MPTP (Wang et al., 2017). Whether chronic occupational exposure to paraquat is causal for PD in humans is debated. Although the connection between paraquat and PD in humans has seemed strong in the past, the more current perspective considers the correlation to be weak (Weed, 2021). Regardless, paraquat has been successfully used for animal models of PD, replicating important hallmarks of the disease such as increased α-synuclein levels and α-synuclein aggregations in SNpc neurons, leading to dopaminergic neuron loss and movement impairment (Cristóvão et al., 2020). hiPSCs that have been differentiated into astrocytes and exposed to paraquat cease to proliferate and have increased levels of SA-β-gal, p16^*INK*4*a*^, and IL-6, indicating a senescent state (Chinta et al., 2018). The hiPSC derived astrocytes also had an increased number of 53BP1 foci due to the upregulation of DNA damage signaling, which stimulates SASP expression (Chinta et al., 2018). Other environmentally toxic chemicals that have been linked to PD, like the pesticide rotenone and 2,3,7,8-tetrachlorodibenzo-p-dioxin (TCDD), have also been shown to cause premature senescence in human astrocytes in a dose-dependent manner (Wan et al., 2014; González-Barbosa et al., 2017; Simmnacher et al., 2020).

The oxidative stress and subsequent inflammation associated with neurodegenerative diseases, non-pathological advanced age, and environmental toxin exposure drive elevated senescent astrocyte levels (Bitto et al., 2010; Si et al., 2021). For example, the hormone angiotensin II has been implicated in producing intracellular free radicals, increasing oxidative stress, promoting mitochondrial dysfunction, and accelerating inflammation related to aging (Benigni et al., 2010). Angiotensin II has also been shown to cause senescence in cultured human astrocytes in a concentration-dependent manner by producing superoxide oxidative stress (Liu et al., 2011). The transcriptome of fetal human astrocytes that have been made senescent by transient oxidative stress exposure have been characterized. Unsurprisingly, genes associated with neural development and differentiation were downregulated as well as some genes related to injury response, whereas pro-inflammatory genes were upregulated (Crowe et al., 2016).

## Interactions Between α-Synucleinopathy and Neuroglial Senescence in Parkinson’s Disease

### A Self-Amplifying and Vicious Cycle

The physiological connections between senescent neuroglia and the development of neuronal α-synucleinopathy are feed-forward oxidative stress and inflammation cascades. The brain is a highly metabolically active organ and consumes about 20% of the basal oxygen in humans (Cobley et al., 2018). Free radicals are abundant in the brain and are necessary for the delicate redox signaling that is critical for many CNS functions (Cobley et al., 2018; Franco and Vargas, 2018; van Leeuwen et al., 2020). However, when the oxidative balance favors cellular stress, cellular damage results. According to the free radical theory of aging, a consequence of aerobic metabolism over the lifespan is oxidative damage that results in the aging process (Harman, 1956).

Central nervous system oxidative stress has been shown to induce neuroglial senescence. Specific oxidative stress effects on neuroglia that lead to a senescent state include morphological changes, disrupted mitochondrial function, altered cellular signaling, altered cellular metabolism, damaged DNA, shortened telomeres, altered chromatin structure, altered proteostasis, and impaired DDR (Correia-Melo and Passos, 2015; Vasileiou et al., 2019; Martínez-Cué and Rueda, 2020). The pro-inflammatory SASP of senescent astrocytes and microglia contribute to the low-grade chronic inflammation that is associated with CNS aging (Norden and Godbout, 2013; Streit et al., 2014; von Bernhardi et al., 2015; Martínez-Cué and Rueda, 2020). CNS inflammation contributes to the α-synuclein toxicity that drives α-synucleinopathy in PD. Neuroinflammation is increasingly being recognized as one of the major contributors to PD pathology rather than just a by-product of the disease process itself (Harms et al., 2021; Hirsch and Standaert, 2021).

Neuroinflammation in PD involves both the innate immune system and the adaptive immune system since PD-related BBB compromise facilitates the entrance of peripheral immune cells into the CNS (de Vries et al., 2012; Cardinale et al., 2021). The two Nod-like receptor (NLR) proteins, NLRP3 and NLRC4, are part of the innate immune system, are inflammasome activators in microglia and astrocytes, and activate caspase-1 (Freeman et al., 2017). NLRP3 activation in microglia by ROS leads to the release of the pro-inflammatory cytokines IL-1β and IL-18 (Kam et al., 2020). ROS can also activate NLRC4 in astrocytes, prompting the release of IL-1β and IL-18 as well (Lim et al., 2019).

Concerning PD pathology, activated caspase-1 cleaves α-synuclein into a truncated form that is highly prone to forming the insoluble aggregates characteristic of Lewy bodies (Wang et al., 2016). There is robust *in vitro* evidence that inflammasome-activated, caspase-1-induced α-synucleinopathy is cytotoxic (Wang et al., 2016; Ma et al., 2018). Caspase-1 has also been shown to truncate α-synuclein at the C-terminal end in the proteolipid protein α-synuclein (PLP-SYN) transgenic mouse model of MSA, which promoted α-synuclein aggregation, motor deficits, and a reduction in tyrosine hydroxylase-positive neurons in the substantia nigra (Bassil et al., 2016). Furthermore, the capase-1 inhibitor VX-765 is neuroprotective in the PLP-SYN mouse model (Bassil et al., 2016). Caspase-like truncation of the C-terminal end of α-synuclein has been discovered and characterized in other PD models of transgenic mice, cell culture, and recombinant adeno-associated virus injected rats (Li et al., 2005; Liu et al., 2005; Ulusoy et al., 2010). Truncated α-synuclein as well as full-length α-synuclein have been widely observed in post-mortem brains from PD patients and patients with Dementia with Lewy bodies (Suzuki et al., 2018). Therefore, when microglia and astrocytes are exposed to age-related chronic oxidative stress, they progress to releasing the pro-inflammatory SASP and convert WT α-synuclein into a PD-related toxic species.

Senescent neuroglia prime neurons for neurodegeneration and contributes to the early pathology. For example, when healthy neurons are co-cultured with senescent neuroglia, they experience a reduction in function, synapse maturation, synaptic plasticity, synaptic vesicle size, and disrupted neuronal homeostasis (Bussian et al., 2018; Han et al., 2020; Limbad et al., 2020; Sheeler et al., 2020). The factors involved in senescent neuroglial mediated neuronal detriment include the pro-inflammatory secretion, reduced neurotrophic factor secretion, reduced glutathione secretion, and the reduced ability to clear extracellular α-synuclein (Rodriguez et al., 2015; Burtscher and Millet, 2021). Furthermore, it has been shown that removing senescent neuroglia from models of neurodegeneration mitigates reactive gliosis and neuronal death while preserving normal organismal physiology (Chinta et al., 2018; Salas et al., 2020).

The degeneration of dopaminergic neurons in PD can initiate a self-propagating cycle of oxidative stress, neuroinflammation, neuroglial senescence, neuroglial activation, and neuronal death. The injured, degenerative dopaminergic neurons in PD release insoluble α-synuclein fibrils, ATP, MMP-3, and neuromelanin into the extracellular space (He et al., 2021). In an effort to maintain brain homeostasis, microglia and astrocytes are prompted to take up the cellular debris from degenerating neurons and initiate the subsequent inflammatory and oxidative stress responses. Although these actions of neuroglia are necessary and beneficial in a non-pathologic state, when they can no longer combat the tide of degeneration, the cycle becomes self-amplifying and destructive.

### The Effects of Neuroglial Uptake of α-Synuclein and Activation by α-Synuclein

Neurons secrete α-synuclein into the extracellular space through an exosomal and calcium-dependent manner (Emmanouilidou et al., 2010). The rate of α-synuclein secretion from neurons and neuroblastomas into the extracellular space, as well as the concentration of secreted insoluble α-synuclein aggregates, increases under cellular stress-induced protein misfolding and damage (Jang et al., 2010). The damaged, aberrant α-synuclein are secreted into the extracellular space under stress through exocytosis rather than in exosomes (Jang et al., 2010). Extracellular wild-type and mutant α-synuclein can be taken up by neurons or glia *via* endocytosis and transmitted between glia and neurons, even in aggregated form, leading to the spread of Lewy bodies (Lee et al., 2011, 2014).

Extracellular non-mutated α-synuclein oligomers elicit an inflammation response from microglia *via* paracrine activation of Toll-like receptor 2 (TLR2) (Kim et al., 2013; Lee et al., 2014). Moreover, extracellular α-synuclein can act as damaged-associated molecular patterns (DAMPs) to activate other microglial receptors and intracellular pathways, such as the Fc gamma receptor IIB (FcγRIIB) and the NF-κB pathway (Kam et al., 2020). The activation of these receptors and pathways lead to reduced microglial phagocytosis, an upregulated inflammation response, α-synuclein nitration and increased release of ROS (Zhang et al., 2007; Kam et al., 2020; Mavroeidi and Xilouri, 2021).

A robust debate over whether activated microglia are harmful or beneficial spans back more than 20 years (Streit et al., 1999). In PD, chronically activated microglia increase neuronal susceptibility for neurodegeneration. Permanently senescent microglia clearly differ from the transiently activated microglia. Microglia become transiently activated in response to brain injury and upregulate both pro-inflammatory and anti-inflammatory genes simultaneously (Osman et al., 2020). However, the chronically activated microglia that are commonly observed in neurodegeneration seem to significantly differ from senescent microglia in semantics only (Lull and Block, 2010; Woodburn et al., 2021). Naturally, there are slight differences between senescent and chronically activated microglia, but they seem to be functionally very similar in PD. We hypothesize that the senescent, chronically activated, or transiently activated fates reflect the heterogeneity of microglial populations and the effects of individual microglial microenvironments (Masuda et al., 2020; Tan et al., 2020). Operating under this hypothesis, it is possible that some senescent microglia adopt a chronically activated phenotype due to an early neurodegenerative milieu and contribute to the further development of the neurodegeneration in a chronic manner (Masuda et al., 2020).

Chronically activated microglia contribute to the early development of PD in response to interacting with α-synuclein and remain chronically activated as long as α-synuclein is present. For example, an *in vivo* mouse model that overexpressed full-length, wild-type, human α-synuclein under the murine Thy-1 promoter experienced lifelong activation of microglia (Chesselet et al., 2012). Activated microglia first appeared in the striata at 1 month old and in the SNpc at about 5 months old, before neuronal damage was observed (Chesselet et al., 2012). People with idiopathic rapid-eye-movement sleep behavior (IRBD) serve as an interesting population for studying the preclinical hallmarks of PD. Nine out of ten people with IRBD receive a PD diagnosis within 14 years of receiving an IRBD diagnosis (Iranzo et al., 2016). The pathology is strikingly similar between the two diseases. For example, striatal ^18^F-DOPA is reduced in 90% of IRBD patients, coupled with unilateral microglia activation in the SNpc, consistent with early PD (Ouchi, 2017). These observations correlate with the notion that inflammation due to chronically activated microglia facilitates the change from IRBP to PD over time. Indeed, postmortem PD brains have substantial quantities of pro-inflammatory microglia and astrocytes in the same areas affected by α-synucleinopathy (Freund et al., 2012; Chinta et al., 2018; Brás et al., 2020; Harms et al., 2021).

Extracellular α-synuclein endocytosed by astrocytes cause the release of proinflammatory and neuroinhibitory secretions, such as GFAP, cytokines, chemokines, and chondroitin sulfate proteoglycan (Vieira et al., 2020). However, there is some evidence that α-synuclein uptake by astrocytes follows α-synuclein binding to astrocyte receptors to stimulate proinflammatory secretions (Vieira et al., 2020). Endocytosed α-synuclein in astrocytes causes disrupted Ca^2+^ and mitochondrial homeostasis, oxidative stress, and elevated levels of glutathione peroxidase (Mavroeidi and Xilouri, 2021). Astrocytes can harbor insoluble α-synuclein aggregates (Lee et al., 2010). The uptake of α-synuclein aggregates by astrocytes is initially a protection mechanism that aims to clear the toxic protein (Booth et al., 2017). However, the pathological inclusion can cause lysosomal dysfunction, can remain in astrocytes, and therefore, accumulate and cause cellular damage (Booth et al., 2017).

Astrocytes have a unique susceptibility to becoming senescent in response to cellular stress. In response to oxidative stress, astrocytes become senescent before fibroblasts, neurons, and possibly microglia (Bitto et al., 2010; Chinta et al., 2018). In postmortem PD SNpc tissue, the only monitored cell type to have a reduction in nuclear lamin B1 compared to control SNpc tissue were astrocytes (Chinta et al., 2018). Reduced lamin B1 levels are a long standing biomarker of cellular senescence (Freund et al., 2012). However, microglia are the first line of defense in response to injury and oxidative stress. They exhibit fast migration to the injured area in order to initiate phagocytosis and quickly become activated. Soon afterward, microglia recruit astrocytes to become activated, release pro-inflammatory factors and promote glutamate-induced excitotoxicity (Liddelow et al., 2017; Iovino et al., 2020; Liu et al., 2020; Matejuk and Ransohoff, 2020). If this same pattern is seen in neurodegeneration-related chronic neuroglial activation, then microglia would become “senescent” first. Even if astrocytes become senescent before their cellular neighbors do, the bystander effect ensures the spread of senescence to other cell types (Nelson et al., 2012).

### The Effects of Neuroglial Exposure to Preformed α-Synuclein Fibrils

There are three morphological species of α-synuclein. From smallest to largest, they are monomers, oligomers, and fibrils. The major pathological components of Lewy Bodies are α-synuclein fibrils. Artificially synthesized “preformed α-synuclein fibrils” (PFFs) can be injected into animal models of PD to study the spatiotemporal dynamics of α-synuclein movement, inflammation, oxidative stress and neurodegeneration. In response to the presence of α-synuclein fibrils, neuroglia generate superoxide (O2-), ROS, and cytotoxic factors (He et al., 2021). PFFs also exert a strong response in dopaminergic neurons. For example, dopaminergic neurons that are treated with synthesized PFFs experience elevated levels of serine 129 phosphorylated α-synuclein, increased α-synuclein aggregation, reduced levels of presynaptic protein, axonal transport protein disruption, and reduced dopaminergic survival (Tapias et al., 2017). These results are attributed to PFF-induced mitochondrial disfunction, increased O2- production, increased nitric oxide (NO) production, levels of protein nitration, and inflammation (Tapias et al., 2017).

In a recent study, PFFs were injected into the striata of healthy, young adult, wild-type (C57BL/6) male mice, which initially caused an inflammation response in both astrocytes and microglia (Lai et al., 2021). The inflammatory state peaked at 7 days post-injection (dpi). Aggregates of α-synuclein then increased in concentration and propagation after fourteen dpi and peaked between thirty and ninety dpi (Lai et al., 2021). Finally, striatal dopaminergic neuron loss and motor disfunction were observed (Lai et al., 2021). Similar results were seen in male Fischer 344 rats that received unilateral intrastriatal injections of PFFs (Duffy et al., 2018). Microglial activation and associated inflammation were the initial responses to PFF injection in the rats (Duffy et al., 2018). Microglial activation in response to the PFF lasted for at least the 3 months before SNpc neuronal degeneration occurred and persisted throughout the degenerative process, indicating that the microglia were chronically activated (Duffy et al., 2018).

In another recent study described here, PFF gave rise to activated neuroglia, senescent neuroglia and neuronal death (Verma et al., 2021). MPP^+^ or PFF treatment of cultured dopaminergic rat N27 cells caused the cells to express senescence markers, such as reduced levels of Lamin B1 and HMGB1 and increased levels of p16 and p21. PFF treatment of cultured primary astrocytes and microglia from C57BL/6 wild-type mice also lead to senescence, as evidenced by decreased Lamnin B1, HMGB1, AT-rich sequence-binding protein 1 (SATB1), and p16 levels, but elevated p21. Interestingly, there was simultaneous production of senescent and reactive astroglia in response to primary culture PFF treatment. This observation might reflect the different subpopulations of astrocytes (Miller, 2018). Mice that received brain PFF injections through cannula demonstrated the same changes in senescent markers. For example, the ventral midbrain and SNpc of these mice experienced reduced levels of Lamnin B1, HMGB1, and p16 levels and increased levels of p21. Increased levels of GFAP and Iba-1 were also seen, indicative of reactive astroglia and microglia respectively. Moreover, there was evidence of neuronal death by reduced levels of β-III-tubulin. Finally, SNpc tissue from postmortem PD patients confirmed the involvement of cellular senescence by western blot analysis. Lamnin B1, HMGB1, and SATB1 were reduced, p21 levels were increased and p16 levels remained unchanged in postmortem PD brains compared to control midbrain tissues. The experimental results from Verma et al. (2021) highlights how pathologic α-synuclein instigates simultaneous neuroglial senescence and neuroglial activation that eventually lead to PD-relevant neuronal death.

## Senolytics As A Therapeutic Avenue for Parkinson’s Disease

Senescent cells contribute to a variety of age-related diseases. Conversely, their removal mitigates their associated pathological effects and increases the healthspan. Reduced activity of the SATB1 protein in dopaminergic neurons has recently been identified as a risk factor for PD (Brichta et al., 2015; Chang et al., 2017; Nalls et al., 2019; Riessland, 2020). Furthermore, genetic knockout of *Satb1* leads to cellular senescence and increased expression of p21 and CDKN1A in human embryonic stem cells that have differentiated into dopaminergic neurons (Riessland et al., 2019). Riessland et al. (2019) also showed this phenomenon in the midbrains of mice by using a stereotactic adeno-associated virus 1 injection expressing shRNA (AAV1-shRNA) to downregulate *Satb1*, which subsequently increased p21 expression and neuronal senescence. Eventually, treatment with AAV1-shRNA eliminated tyrosine hydroxylase expressing neurons, reduced the number of mitochondria, upregulated *Cdkn1a*, and prompted an immune response (Riessland et al., 2019). Additionally, postmortem SNpc tissue from PD patients have elevated p21 expression and decreased regulatory function of SATB1 (Brichta et al., 2015; Riessland et al., 2019; Riessland, 2020). Inhibition of p21 in SATB1 knockout human dopaminergic neurons *via* the p21 inhibitor UC2288 significantly reduced the effects of senescence without producing proliferation (Riessland et al., 2019). Furthermore, treatment of SATB1 knockout human dopaminergic neurons with CDKN1A short hairpin RNA (shRNA) dramatically reduced p21 levels and other senescence hallmarks (Riessland et al., 2019). Additionally, UC2288 has recently been shown to reduce senescence markers such as oxidative stress and inflammation in the MPTP mouse model of PD (Im et al., 2020). Therefore, UC2288 might be a viable anti-senescent agent for PD.

Astragaloside IV (AS-IV) is the active pharmacological agent derived from the herbal plant Astragalus membranaceus. AS-IV has a long history in Chinese herbal medicine due to its many beneficial properties, such as being a powerful antioxidant, antifibrotic and anti-inflammatory agent (Li et al., 2017). AS-IV is neuroprotective in primary dopaminergic nigral cell culture exposed to 6-hydroxydopamine (Chan et al., 2009). Mice treated with chronic MPTP and probenecid injections experience a significant loss of dopaminergic neurons in the SNpc and suffer from loss of muscle strength and balance (Xia et al., 2020). However, when receiving co-treatment with AS-IV, dopaminergic neurons and motor deficits in MPTP and probenecid treated mice have significant protection without altering MPTP metabolism (Xia et al., 2020). An important finding from the Xia et al. (2020) study was that AS-IV treatment reduced the SNpc concentration of senescent astrocytes in the MPTP mouse model and improved many markers of cellular senescence, such as elevated p16 levels and reduced levels of lamin B1 in the cellular nucleus. Furthermore, natural age-related senescence and premature senescence due to MPP + treatment in primary astrocyte culture from mice were shown to be inhibited by the AS-IV treatment (Xia et al., 2020). AS-IV was shown to exert its anti-senescent effect through promoting mitophagy and its antioxidant properties (Xia et al., 2020).

It has recently been shown that astrocyte and microglia senescence in PD can be mitigated by senolytic treatment with the serum and glucocorticoid related kinase 1 (SGK1) inhibitor GSK-650394 (Kwon et al., 2021). NF-kB transcription factors are responsible for transcribing pro-inflammatory genes, including those for cytokines and chemokines (Liu et al., 2017). Through phosphorylation, SGK1 activates NF-kB pathways and promotes inflammatory responses (Lang and Voelkl, 2013). GSK-650394 reduces cytokine levels and SGK1 overexpression boosted cytokine levels in cultured mice astrocytes and microglia from the cortex and ventral midbrain (Kwon et al., 2021). Furthermore, Nurr1 and Foxa2 downregulate *Sgk1* in the mouse cultured glia, as shown in microarray and RNA-seq data (Kwon et al., 2021). Seven out of the top ten genes that were downregulated by GSK-650394 had immune-related ontologies (Kwon et al., 2021). Therefore, the anti-inflammatory effects of Nurr1 and Foxa2 in glia are due to inhibitory action on *Sgk1*.

It has also been shown that SGK1 inhibition suppresses inflammation pathways associated with the NLRP3 inflammasome and CGAS-STING, upregulates glutamate clearance from glia, and prevents glial mitochondrial damage (Kwon et al., 2021). Finally, SGK1 inhibition reduced glial senescent markers such as SA-β-gal, downregulated genes associated with SASP, reduced pro-senescent protein levels, reduced reactive oxygen species production, and downregulated pro-oxidant genes (Kwon et al., 2021). Importantly, mouse midbrain dopaminergic neurons that overexpressed human α-synuclein were co-cultured with mouse ventral midbrain astrocytes and microglia. These cultures were treated with PFFs. Culture treatment with GSK-650394 or SGK1 knockdown in the glia reduced α-synuclein pathology in neurons including α-synuclein neuron-to-neuron transfer, provided they were co-cultured with the ventral midbrain glia (Kwon et al., 2021). Furthermore, SGK1 inhibition in mouse ventral midbrain astrocytes and microglia co-cultured with mouse midbrain dopaminergic neurons protected the neurons from toxic insult from H_2_O_2_. Finally, SGK1 genetic silencing or GSK-650394-mediated inhibition in the MPTP mouse model of PD protected against behavioral deficits, midbrain dopaminergic neurons loss, and suppressed SNpc inflammation and senescence (Kwon et al., 2021).

B-cell lymphoma-extra large (Bcl-xL) is a member of the Bcl-2 protein family and resides in mitochondrial membranes. Bcl-xL has anti-apoptotic properties mediated through its inhibition of mitochondrial cytochrome c release (D’Aguanno and Del Bufalo, 2020). Furthermore, Bcl-xL also has pro-senescent properties. For example, it is hypothesized that damaged cells otherwise destined for apoptosis can instead become senescent through the overexpression of Bcl-xL (Mas-Bargues et al., 2021). Bcl-xL also enhances mitochondrial metabolism and increases the efficiency of ATP synthesis, both of which are necessary to metabolically support the increased SASP production of senescent cells (Herranz and Gil, 2018; Mas-Bargues et al., 2021).

In postmortem brain samples from PD patients, it was shown that Bcl-xL expression in mesencephalon dopaminergic neurons was close to twice as high as in the controls (Hartmann et al., 2002). Interestingly, Bcl-xL is likely involved in sporadic PD through pro-senescence and anti-Parkin activity. Under normal conditions, the PINK1 protein cooperates with the protein Parkin to translocate to polarized mitochondria and induce mitophagy. However, mutated E3 ubiquitin ligase Parkin and pathological mitochondrial bioenergetics are implicated in autosomal recessive familial PD (Dawson and Dawson, 2010). It has been shown that Bcl-xL antagonizes the ability of PINK1 and Parkin to stimulate mitophagy (Mas-Bargues et al., 2021). Disrupted midbrain mitophagy is a foundational pathological feature common in both PD patients and PD animal models (Liu et al., 2019). Therefore, it seems likely that Bcl-xL inhibitors, such as A1331852 and A1155463, could be effective therapeutic agents in PD through promoting mitophagy (Zhu et al., 2017).

The relationship between PD pathology and Bcl-xL is complicated. Like cellular senescence, Bcl-xL seems to have the capacity in PD for neuroprotection, as well as exacerbating pathology. For example, SH-SY5Y cells transfected with a dopamine transporter were resistant to MPP^+^ when treated with Bcl-xL (Dietz et al., 2008). SH-SY5Y cells overexpressing Bcl-xL were also resistant to 6-hydroxydopamine induced death (Jordán et al., 2004). Additionally, SH-SY5Y cells overexpressing Bcl-xL preserved mitochondrial dynamics through anti-oxidative stress mechanisms when LRRK2 was pharmacologically inhibited by GSK2578215A (Saez-Atienzar et al., 2016). Furthermore, Bcl-xL treatment was shown to be neuroprotective in the MPTP mouse model of PD (Dietz et al., 2008). Finally, Bcl-xL is necessary for CNS synapse formation, synaptic vesicle membrane dynamics, and neurite outgrowth, all of which become disrupted during neurodegeneration (Li et al., 2008, 2013; Park et al., 2015). Although these results were only demonstrated in cell culture and imperfect mouse models of PD, they do raise some hesitancy for pursuing senolytic Bcl-xL antagonists as a therapeutic avenue.

Despite the potential dual role of Bcl-xL in PD, perhaps the different effects can be parsed and capitalized on. The Bcl-xL protein can be cleaved at its N-terminus by caspase-dependent mechanisms to produce ΔN-Bcl-xL fragments. Bcl-xL fragmentation is increased during glutamate-induced neuroexitotoxicity, which commonly occurs in many neurodegenerative diseases, including PD (Park and Jonas, 2017; Iovino et al., 2020). Accumulation of ΔN-Bcl-xL fragments induces mitochondrial injury, such as elevated membrane conductance and increased cytochrome c release, eventually leading to neuronal death (Park and Jonas, 2017). The senolytic ABT-737 binds to both Bcl-xL and ΔN-Bcl-xL, prevents ΔN-Bcl-xL from damaging mitochondria, and prevents the Bcl-xL from forming ΔN-Bcl-xL fragments (Park and Jonas, 2017). Furthermore, it has been shown that the effects of Bcl-xL senolytics are concentration-dependent. For example, high concentrations of ABT-737 (1 μM) and WEHI-539 (5 μM) exacerbated neurotoxicity from glutamate, disrupts mitochondria membrane potential, and reduces the cellular concentration of ATP (Park et al., 2017). Conversely, a low concentration of ABT-737 (10 ηM) and WEHI-539 (10 ηM) was neuroprotective against glutamate-induced cell death through protecting mitochondrial membrane potential and preserving ATP loss (Park et al., 2017). Together, the evidence seems to suggest that Bcl-xL still holds promise as a target in the anti-senescence treatment of PD, but the concentration of Bcl-xL specific senolytics and Bcl-xL fragmentation potential needs to be taken into consideration. Since Bcl-xL fragmentation occurs in response to glutamate neurotoxicity, perhaps Bcl-xL specific senolytics would have more effect before disease onset.

Anti-senescent or senescent cell removal strategies seem like a possible new avenue of pharmacological therapy for PD patients. However, the majority of evidence is currently limited and restricted to cell and animal models as described in Table 1. Indeed, the majority of therapeutic studies of senolytics are pre-clinical (Romashkan et al., 2021). However, the first open-label, single-arm clinical trial of senolytics in human patients was published in 2019 (Justice et al., 2019; Song et al., 2020). This study showed that short-term (3 weeks) treatment of senolytic cells in patients with idiopathic pulmonary fibrosis with dasatinib and quercetin (D + Q) improved symptoms and function (Justice et al., 2019; Song et al., 2020). Since then, D + Q has also been shown to be effective at decreasing senescent cells in diabetic kidney disease patients (Hickson et al., 2019). There has been a rapid increase in the number and scope of the clinical trials centered on senolytics just during this past year (Kirkland and Tchkonia, 2020; Song et al., 2020; Wissler Gerdes et al., 2020). Concurrently, there has also been a rapid rise in pharmaceutical companies and capitalist investments focused exclusively on developing senolytics over the last handful of years (Dolgin, 2020).

**TABLE 1:** Anti-senescent drugs that have therapeutic potential relevant for Parkinson’s disease.

Senolytic	Evidence	Citation
UC2288	Reduces neuronal senescence in MPTP mouse model and primary cell culture through inhibiting p21, oxidative stress, and inflammation	Riessland et al., 2019
Astragaloside IV	Removed senescent astrocytes in MPTP mouse model and MPP^+^ cell culture through promoting mitophagy and antioxidant properties	Xia et al., 2020
GSK-650394	Inhibits astrocyte and microglia SGK1 mediated neuronal senescence and inflammation	Kwon et al., 2021
ABT-737 and WEHI-539	Low concentrations inhibit Bcl-xL fragmentation and ΔN-Bcl-xL mediated neuronal damage	Park et al., 2017

There are fourteen clinical studies currently listed at ClinicalTrials.gov that result from searching “senolytic” in the “other” search field. Four trials target osteoarthritis, four are focused on mitigating COVID-19, and the rest center on femoroacetabular impingement, frailty in adult survivors of childhood cancer, chronic kidney disease, and improving the skeletal health of healthy older adults. In a variety of combinations and dosings, the senolytics D, Q, and fisetin are included as drug interventions in all of these clinical trials. One of the osteoarthritis trials includes fisetin and also the antihypertensive drug losartan. Another current osteoarthritis trial includes Q, fisetin, and also glycyrrhizin as interventions. Glycyrrhizin has anti-inflammatory and antiviral properties. Previous or planned senolytic focused clinical trials have employed their use in the treatment of hyperoxia-induced reactive airway disease, insulin resistance, diabetes, pre-eclampsia, fatty liver disease, obesity, macular degeneration, and diabetic chronic kidney disease (Kirkland and Tchkonia, 2020; Song et al., 2020).

Out of the fourteen results, only two trials focus on neurodegeneration: a pilot and phase II trial of the SToMP-AD study (Senolytic Therapy to Modulate the Progression of Alzheimer’s disease). The pilot study (ClinicalTrials.gov Identifier: NCT04063124) focuses on the use of D + Q for five patients with early-stage Alzheimer’s disease over a 12-week period (Gonzales et al., 2022). The Phase II SToMP-AD study is currently recruiting and plans to include both patients with Alzheimer’s disease and Mild Cognitive Impairment (ClinicalTrials.gov Identifier: NCT04685590). It is fully expected that effective senolytic-based therapy for PD patients and their families will be a reality in the not-too-distant future.

## Conclusion

Parkinson’s disease is the most common movement disorder and the second most common neurodegenerative disorder. However, the complex pathology is not yet fully understood nor is there a cure available. The study of PD has largely focused on neurons since the disease is marked by progressive neurodegeneration. PD research has also largely centered on aggregated α-synuclein since they are the key molecular hallmark of the disease. However, neuroglia account for a large portion of the brain and are responsible for a myriad of critical functions in the CNS. The roles of neuroglia in neurodegenerative diseases are underappreciated. Targeting senescent neuroglia in PD is an exciting possible therapeutic avenue. Clinical trials of anti-senescent drugs have very recently started to get underway and hold much promise.

## Author Contributions

All authors contributed to the writing of the manuscript, have reviewed the manuscript, and approve of its current form. RL oversaw the scope and progress of the manuscript, wrote the final draft, and made the final figures.

## Conflict of Interest

SM and RL are employed by Pluripotent Diagnostics. The remaining authors declare that the research was conducted in the absence of any commercial or financial relationships that could be construed as a potential conflict of interest.

## Publisher’s Note

All claims expressed in this article are solely those of the authors and do not necessarily represent those of their affiliated organizations, or those of the publisher, the editors and the reviewers. Any product that may be evaluated in this article, or claim that may be made by its manufacturer, is not guaranteed or endorsed by the publisher.

## References

[B1] Abate M. Festa A. Falco M. Lombardi A. Luce A. Grimaldi A. (2020). Mitochondria as playmakers of apoptosis, autophagy and senescence. *Semin. Cell Dev. Biol.* 98 139–153. 10.1016/j.semcdb.2019.05.022 31154010

[B2] Acosta-Martínez M. (2020). Shaping microglial phenotypes through estrogen receptors: relevance to sex-specific neuroinflammatory responses to brain injury and disease. *J. Pharmacol. Exp. Therapeut.* 375 223–236. 10.1124/jpet.119.264598 32513838

[B3] Angelova D. M. Brown D. R. (2019). Microglia and the aging brain: are senescent microglia the key to neurodegeneration? *J. Neurochem.* 151 676–688. 10.1111/jnc.14860 31478208

[B4] Arrasate M. Finkbeiner S. (2012). Protein aggregates in Huntington’s disease. *Exp. Neurol.* 238 1–11. 10.1016/j.expneurol.2011.12.013 22200539 PMC3909772

[B5] Bachiller S. Jiménez-Ferrer I. Paulus A. Yang Y. Swanberg M. Deierborg T. (2018). Microglia in neurological diseases: a road map to brain-disease dependent-inflammatory response. *Front. Cell. Neurosci.* 12:1–17. 10.3389/fncel.2018.00488 30618635 PMC6305407

[B6] Baker D. J. Petersen R. C. (2018). Cellular senescence in brain aging and neurodegenerative diseases: evidence and perspectives. *J. Clin. Investig.* 128 1208–1216. 10.1172/JCI95145 29457783 PMC5873891

[B7] Bakshi R. Logan R. Schwarzschild M. A. (2015). “Purines in parkinson’s: Adenosine A2A receptors and urate as targets for neuroprotection,” in *The Adenosinergic System: A Non-Dopaminergic Target in Parkinson’s Disease*, eds Morelli M. Simola N. Wardas J. (Berlin: Springer), 10.1007/978-3-319-20273-0_6

[B8] Barkholt P. Sanchez-Guajardo V. Kirik D. Romero-Ramos M. (2012). Long-term polarization of microglia upon α-synuclein overexpression in nonhuman primates. *Neuroscience* 208 85–96. 10.1016/j.neuroscience.2012.02.004 22342967

[B9] Bartels T. Ahlstrom L. S. Leftin A. Kamp F. Haass C. Brown M. F. (2010). The N-terminus of the intrinsically disordered protein α-synuclein triggers membrane binding and helix folding. *Biophys. J.* 99 2116–2124. 10.1016/j.bpj.2010.06.035 20923645 PMC3042581

[B10] Bartels T. Choi J. G. Selkoe D. J. (2011). α-Synuclein occurs physiologically as a helically folded tetramer that resists aggregation. *Nature* 477 107–111. 10.1038/nature10324 21841800 PMC3166366

[B11] Bassil F. Fernagut P. Bezard E. Pruvost A. Leste-lasserre T. Hoang Q. Q. (2016). Reducing C-terminal truncation mitigates synucleinopathy and neurodegeneration in a transgenic model of multiple system atrophy. *Proc. Natl. Acad. Sci. USA.* 113 9593–9598. 10.1073/pnas.1609291113 27482103 PMC5003293

[B12] Bendor J. T. Logan T. P. Edwards R. H. (2013). The function of α-synuclein. *Neuron* 79 1044–1066. 10.1016/j.neuron.2013.09.004 24050397 PMC3866954

[B13] Benigni A. Cassis P. Remuzzi G. (2010). Angiotensin II revisited: new roles in inflammation, immunology and aging. *EMBO Mol. Med.* 2 247–257. 10.1002/emmm.201000080 20597104 PMC3377325

[B14] Bertoncini C. W. Fernandez C. O. Griesinger C. Jovin T. M. Zweckstetter M. (2005). Familial mutants of α-synuclein with increased neurotoxicity have a destabilized conformation. *J. Biol. Chem.* 280 30649–30652. 10.1074/jbc.C500288200 16020550

[B15] Bhat R. Crowe E. P. Bitto A. Moh M. Katsetos C. D. Garcia F. U. (2012). Astrocyte senescence as a component of alzheimer’s disease. *PLoS One* 7:1–10. 10.1371/journal.pone.0045069 22984612 PMC3440417

[B16] Bick R. J. Poindexter B. J. Kott M. M. Liang Y. Dinh K. Kaur B. (2008). Cytokines disrupt intracellular patterns of Parkinson’s disease-associated proteins alpha-synuclein, tau and ubiquitin in cultured glial cells. *Brain Res.* 1217 203–212. 10.1016/j.brainres.2008.03.081 18501880

[B17] Birch J. Gil J. (2020). Senescence and the SASP: many therapeutic avenues. *Genes Dev.* 34 1565–1576. 10.1101/gad.343129.120 33262144 PMC7706700

[B18] Bitto A. Sell C. Crowe E. Lorenzini A. Malaguti M. Hrelia S. (2010). Stress-induced senescence in human and rodent astrocytes. *Exp. Cell Res.* 316 2961–2968. 10.1016/j.yexcr.2010.06.021 20620137

[B19] Blauwendraat C. Nalls M. A. Singleton A. B. (2020). The genetic architecture of Parkinson’s disease. *Lancet Neurol.* 19 170–178. 10.1016/S1474-4422(19)30287-X 31521533 PMC8972299

[B20] Bliederhaeuser C. Grozdanov V. Speidel A. Zondler L. Ruf W. P. Bayer H. (2016). Age-dependent defects of alpha-synuclein oligomer uptake in microglia and monocytes. *Acta Neuropathol.* 131 379–391. 10.1007/s00401-015-1504-2 26576561

[B21] Blokhuis A. M. Groen E. J. N. Koppers M. Van Den Berg L. H. Pasterkamp R. J. (2013). Protein aggregation in amyotrophic lateral sclerosis. *Acta Neuropathol.* 125 777–794. 10.1007/s00401-013-1125-6 23673820 PMC3661910

[B22] Bogale T. A. Faustini G. Longhena F. Mitola S. Pizzi M. Bellucci A. (2021). Alpha-Synuclein in the Regulation of Brain Endothelial and Perivascular Cells: gaps and Future Perspectives. *Front. Immunol.* 12:1–15. 10.3389/fimmu.2021.611761 33679750 PMC7933041

[B23] Book A. Guella I. Candido T. Brice A. Hattori N. Jeon B. (2018). A meta-analysis of α-synuclein multiplication in familial parkinsonism. *Front. Neurol.* 9:1–9. 10.3389/fneur.2018.01021 30619023 PMC6297377

[B24] Booth H. D. E. Hirst W. D. Wade-Martins R. (2017). The role of astrocyte dysfunction in parkinson’s disease pathogenesis. *Trends Neurosci.* 40 358–370. 10.1016/j.tins.2017.04.001 28527591 PMC5462417

[B25] Borodkina A. V. Deryabin P. I. Giukova A. A. Nikolsky N. N. (2018). “Social life” of senescent cells: what is SASP and why study it? *Acta Nat.* 10 4–14. 10.32607/20758251-2018-10-1-4-14 PMC5916729 29713514

[B26] Braak H. Del Tredici K. (2017). Neuropathological staging of brain pathology in sporadic parkinson’s disease: separating the wheat from the chaff. *J. Parkinson’s Dis.* 7 S73–S87. 10.3233/JPD-179001 28282810 PMC5345633

[B27] Braak H. Sandmann-Keil D. Gai W. Braak E. (1999). Extensive axonal Lewy neurites in Parkinson’s disease: a novel pathological feature revealed by α-synuclein immunocytochemistry. *Neurosci. Lett.* 265 67–69. 10.1016/S0304-3940(99)00208-6 10327208

[B28] Braak H. Sastre M. Del Tredici K. (2007). Development of α-synuclein immunoreactive astrocytes in the forebrain parallels stages of intraneuronal pathology in sporadic Parkinson’s disease. *Acta Neuropathol.* 114 231–241. 10.1007/s00401-007-0244-3 17576580

[B29] Brás I. C. Dominguez-Meijide A. Gerhardt E. Koss D. Lázaro D. F. Santos P. I. (2020). Synucleinopathies: where we are and where we need to go. *J. Neurochem.* 153 433–454. 10.1111/jnc.14965 31957016

[B30] Brawek B. Schwendele B. Riester K. Kohsaka S. Lerdkrai C. Liang Y. (2014). Impairment of in vivo calcium signaling in amyloid plaque-associated microglia. *Acta Neuropathol.* 127 495–505. 10.1007/s00401-013-1242-2 24407428

[B31] Brawek B. Skok M. Garaschuk O. (2021). Changing functional signatures of microglia along the axis of brain aging. *Int. J. Mol. Sci.* 22 1–22. 10.3390/ijms22031091 33499206 PMC7865559

[B32] Brichta L. Shin W. Jackson-Lewis V. Blesa J. Yap E. L. Walker Z. (2015). Identification of neurodegenerative factors using translatome-regulatory network analysis. *Nat. Neurosci.* 18 1325–1333. 10.1038/nn.4070 26214373 PMC4763340

[B33] Burmann B. M. Gerez J. A. Matečko-Burmann I. Campioni S. Kumari P. Ghosh D. (2020). Regulation of α-synuclein by chaperones in mammalian cells. *Nature* 577 127–132. 10.1038/s41586-019-1808-9 31802003 PMC6930850

[B34] Burtscher J. Millet G. P. (2021). Hypoxia, Acidification and Inflammation: partners in Crime in Parkinson’s Disease Pathogenesis? *Immuno* 1 78–90. 10.3390/immuno1020006

[B35] Bussian T. J. Aziz A. Meyer C. F. Swenson B. L. van Deursen J. M. Baker D. J. (2018). Clearance of senescent glial cells prevents tau-dependent pathology and cognitive decline. *Nature* 562 578–582. 10.1038/s41586-018-0543-y 30232451 PMC6206507

[B36] Cardinale A. Calabrese V. de Iure A. Picconi B. (2021). Alpha-synuclein as a prominent actor in the inflammatory synaptopathy of parkinson’s disease. *Int. J. Mol. Sci.* 22:22126517. 10.3390/ijms22126517 34204581 PMC8234932

[B37] Cerri S. Mus L. Blandini F. (2019). Parkinson ‘ s Disease in Women and Men: what’s the Difference? *J. Parkinson’s Dis.* 9 501–515. 10.3233/JPD-191683 31282427 PMC6700650

[B38] Chai C. Lim K. (2013). Genetic Insights into Sporadic Parkinson’s Disease Pathogenesis. *Curr. Genomics* 14 486–501. 10.2174/1389202914666131210195808 24532982 PMC3924245

[B39] Chan W. S. Durairajan S. S. K. Lu J. H. Wang Y. Xie L. X. Kum W. F. (2009). Neuroprotective effects of Astragaloside IV in 6-hydroxydopamine-treated primary nigral cell culture. *Neurochem. Int.* 55 414–422. 10.1016/j.neuint.2009.04.012 19409437

[B40] Chang D. Nalls M. A. Hallgrímsdóttir I. B. Hunkapiller J. Brug M. van der (2017). A meta-analysis of genome-wide association studies identifies 17 new Parkinson’s disease risk loci. *Nat. Genet.* 49 1511–1516. 10.1038/ng.3955 28892059 PMC5812477

[B41] Chartier-Harlin M.-C. Kachergus J. Roumier C. Mouroux V. Douay X. Lincoln S. (2004). Alpha-synuclein locus duplication as a cause of familial Parkinson’s disease. *Lancet* 364 1167–1169. 10.1016/S0140-6736(04)17103-1 15451224

[B42] Cheignon C. Tomas M. Bonnefont-Rousselot D. Faller P. Hureau C. Collin F. (2018). Oxidative stress and the amyloid beta peptide in Alzheimer’s disease. *Redox Biol.* 14 450–464. 10.1016/j.redox.2017.10.014 29080524 PMC5680523

[B43] Chen X. Burdett T. C. Desjardins C. A. Logan R. Cipriani S. Xu Y. (2013). Disrupted and transgenic urate oxidase alter urate and dopaminergic neurodegeneration. *PNAS* 110:1217296110. 10.1073/pnas.1217296110 23248282 PMC3538212

[B44] Chen X. Chen H. Cai W. Maguire M. Ya B. Zuo F. (2017). The melanoma-linked “redhead” MC1R influences dopaminergic neuron survival. *Ann. Neurol.* 81:24852. 10.1002/ana.24852 28019657 PMC6085083

[B45] Chen X. Umeh C. C. Tainsh R. E. Feng D. D. Maguire M. Zuo F. (2018). Dissociation between urate and blood pressure in mice and in people with early Parkinson’s disease. *EBioMedicine* 37:39. 10.1016/j.ebiom.2018.10.039 30415890 PMC6284456

[B46] Chesselet M. F. Richter F. Zhu C. Magen I. Watson M. B. Subramaniam S. R. (2012). A Progressive Mouse Model of Parkinson’s Disease: the Thy1-aSyn (“Line 61”) Mice. *Neurotherapeutics* 9 297–314. 10.1007/s13311-012-0104-2 22350713 PMC3337020

[B47] Chinta S. J. Woods G. Demaria M. Rane A. Zou Y. McQuade A. (2018). Cellular Senescence Is Induced by the Environmental Neurotoxin Paraquat and Contributes to Neuropathology Linked to Parkinson’s Disease. *Cell Rep.* 22 930–940. 10.1016/j.celrep.2017.12.092 29386135 PMC5806534

[B48] Choi I. Zhang Y. Seegobin S. P. Pruvost M. Wang Q. Purtell K. (2020). Microglia clear neuron-released α-synuclein via selective autophagy and prevent neurodegeneration. *Nat. Commun.* 11:15119–w. 10.1038/s41467-020-15119-w 32170061 PMC7069981

[B49] Cobley J. N. Fiorello M. L. Bailey D. M. (2018). 13 Reasons Why the Brain Is Susceptible To Oxidative Stress. *Redox Biol.* 15 490–503. 10.1016/j.redox.2018.01.008 29413961 PMC5881419

[B50] Cohen J. Torres C. (2019). Astrocyte senescence: evidence and significance. *Aging Cell* 18 1–14. 10.1111/acel.12937 30815970 PMC6516680

[B51] Colla E. (2019). Linking the endoplasmic reticulum to Parkinson’s disease and alpha-synucleinopathy. *Front. Neurosci.* 13:1–10. 10.3389/fnins.2019.00560 31191239 PMC6550095

[B52] Coppé J. P. Patil C. K. Rodier F. Sun Y. Muñoz D. P. Goldstein J. (2008). Senescence-associated secretory phenotypes reveal cell-nonautonomous functions of oncogenic RAS and the p53 tumor suppressor. *PLoS Biol.* 6:60301. 10.1371/journal.pbio.0060301 19053174 PMC2592359

[B53] Coppé J. P. Rodier F. Patil C. K. Freund A. Desprez P. Y. Campisi J. (2011). Tumor suppressor and aging biomarker p16 INK4a induces cellular senescence without the associated inflammatory secretory phenotype. *J. Biol. Chem.* 286 36396–36403. 10.1074/jbc.M111.257071 21880712 PMC3196093

[B54] Correia-Melo C. Passos J. F. (2015). Mitochondria: are they causal players in cellular senescence? *Biochim. Biophys. Acta Bioenerg.* 1847 1373–1379. 10.1016/j.bbabio.2015.05.017 26028303

[B55] Costa J. Martins S. Ferreira P. A. Cardoso A. M. S. Guedes J. R. Peça J. (2021). The old guard: age-related changes in microglia and their consequences. *Mechan. Ageing Dev.* 197:111512. 10.1016/j.mad.2021.111512 34022277

[B56] Cristóvão A. C. Campos F. L. Je G. Esteves M. Guhathakurta S. Yang L. (2020). Characterization of a Parkinson’s disease rat model using an upgraded paraquat exposure paradigm. *Eur. J. Neurosci.* 52 3242–3255. 10.1111/ejn.14683 31958881

[B57] Crowe E. P. Tuzer F. Gregory B. D. Donahue G. Gosai S. J. Cohen J. (2016). Changes in the transcriptome of human astrocytes accompanying oxidative stress-induced senescence. *Front. Aging Neurosci.* 8:1–13. 10.3389/fnagi.2016.00208 27630559 PMC5005348

[B58] Cuollo L. Antonangeli F. Santoni A. Soriani A. (2020). The Senescence-Associated Secretory Phenotype (SASP) in the challenging future of cancer therapy and age-related diseases. *Biology* 9 1–16. 10.3390/biology9120485 33371508 PMC7767554

[B59] D’Aguanno S. Del Bufalo D. (2020). Inhibition of Anti-Apoptotic Bcl-2 Proteins in Preclinical and Clinical Studies: current Overview in Cancer. *Cells* 9:cells9051287. 10.3390/cells9051287 32455818 PMC7291206

[B60] Damani M. R. Zhao L. Fontainhas A. M. Amaral J. Fariss R. N. Wong W. T. (2011). Age-related alterations in the dynamic behavior of microglia. *Aging Cell* 10 263–276. 10.1111/j.1474-9726.2010.00660.x 21108733 PMC3056927

[B61] Dawson T. M. Dawson V. L. (2010). The role of parkin in familial and sporadic Parkinson’s disease. *Mov. Disord.* 25(Suppl. 1), 32–39. 10.1002/mds.22798 20187240 PMC4115293

[B62] De Biase L. M. Schuebel K. E. Fusfeld Z. H. Jair K. Hawes I. A. Cimbro R. (2017). Local cues establish and maintain region-specific phenotypes of basal ganglia microglia. *Neuron* 95 341.e–356.e. 10.1016/j.neuron.2017.06.020 28689984 PMC5754189

[B63] de Vries H. E. Kooij G. Frenkel D. Georgopoulos S. Monsonego A. Janigro D. (2012). Inflammatory events at blood-brain barrier in neuroinflammatory and neurodegenerative disorders: implications for clinical disease. *Epilepsia* 53(Suppl. 6), 45–52. 10.1111/j.1528-1167.2012.03702.x 23134495 PMC4853914

[B64] Del Moral M. O. Asavapanumas N. Uzcátegui N. L. Garaschuk O. (2019). Healthy brain aging modifies microglial calcium signaling in vivo. *Int. J. Mol. Sci.* 20:20030589. 10.3390/ijms20030589 30704036 PMC6386999

[B65] Delamarre A. Meissner W. G. (2017). Epidemiology, environmental risk factors and genetics of Parkinson’s disease. *La Presse Medicale* 46 175–181. 10.1016/j.lpm.2017.01.001 28189372

[B66] Dettmer U. Newman A. J. Von Saucken V. E. Bartels T. Selkoe D. (2015). KTKEGV repeat motifs are key mediators of normal α-synuclein tetramerization: their mutation causes excess monomers and neurotoxicity. *Proc. Natl. Acad. Sci. USA.* 112 9596–9601. 10.1073/pnas.1505953112 26153422 PMC4534262

[B67] Di Leonardo A. Linke S. P. Clarkin K. Wahl G. M. (1994). DNA damage triggers a prolonged p53-dependent G1 arrest and long-term induction of Cip1 in normal human fibroblasts. *Genes Dev.* 8 2540–2551. 10.1101/gad.8.21.2540 7958916

[B68] Dietz G. P. H. Stockhausen K. V. Dietz B. Falkenburger B. H. Valbuena P. Opazo F. (2008). Membrane-permeable Bcl-xL prevents MPTP-induced dopaminergic neuronal loss in the substantia nigra. *J. Neurochem.* 104 757–765. 10.1111/j.1471-4159.2007.05028.x 17995935

[B69] Ding Z. Bin, Song L. J. Wang Q. Kumar G. Yan Y. Q. (2021). Astrocytes: a double-edged sword in neurodegenerative diseases. *Neural Regen. Res.* 16 1702–1710. 10.4103/1673-5374.306064 33510058 PMC8328766

[B70] Dolgin E. (2020). Send in the senolytics. *Nat. Biotechnol.* 38 1371–1377. 10.1038/s41587-020-00750-1 33184478

[B71] Duffy M. F. Collier T. J. Patterson J. R. Kemp C. J. Luk K. C. Tansey M. G. (2018). Lewy body-like alpha-synuclein inclusions trigger reactive microgliosis prior to nigral degeneration. *J. Neuroinflamm.* 15 1–18. 10.1186/s12974-018-1202-9 29716614 PMC5930695

[B72] Eftekharzadeh B. Daigle J. G. Kapinos L. E. Coyne A. Schiantarelli J. Carlomagno Y. (2018). Tau Protein Disrupts Nucleocytoplasmic Transport in Alzheimer’s Disease. *Neuron* 99 925.e–940.e. 10.1016/j.neuron.2018.07.039 30189209 PMC6240334

[B73] Elmore S. (2007). Apoptosis: a Review of Programmed Cell Death. *Toxicol. Pathol.* 35 495–516. 10.1080/01926230701320337 17562483 PMC2117903

[B74] Emmanouilidou E. Melachroinou K. Roumeliotis T. Garbis S. D. Ntzouni M. Margaritis L. H. (2010). Cell-produced α-synuclein is secreted in a calcium-dependent manner by exosomes and impacts neuronal survival. *J. Neurosci.* 30 6838–6851. 10.1523/JNEUROSCI.5699-09.2010 20484626 PMC3842464

[B75] Fanning S. Selkoe D. Dettmer U. (2020). Parkinson’s disease: proteinopathy or lipidopathy? *NPJ Parkinson’s Dis.* 6 1–9. 10.1038/s41531-019-0103-7 31909184 PMC6941970

[B76] Farrer M. Kachergus J. Forno L. Lincoln S. Wang D. Hulihan M. (2004). Comparison of kindreds with parkinsonism and alpha-synuclein genomic multiplications. *Ann. Neurol.* 55 174–179. 10.1002/ana.10846 14755720

[B77] Fellner L. Irschick R. Schanda K. Reindl M. Klimaschewski L. Poewe W. (2013). Toll-like receptor 4 is required for α-synuclein dependent activation of microglia and astroglia. *Glia* 61 349–360. 10.1002/glia.22437 23108585 PMC3568908

[B78] Foulds P. G. Mitchell J. D. Parker A. Turner R. Green G. Diggle P. (2011). Phosphorylated alpha-synuclein can be detected in blood plasma and is potentially a useful biomarker for Parkinson’s disease. *FASEB J.* 25 4127–4137. 10.1096/fj.10-179192 21865317

[B79] Franco R. Vargas M. R. (2018). Redox biology in neurological function, dysfunction, and aging. *Antioxid. Redox Signal.* 28 1583–1586. 10.1089/ars.2018.7509 29634346 PMC5962327

[B80] Freeman L. Guo H. David C. N. Brickey W. J. Jha S. Ting J. P. Y. (2017). NLR members NLRC4 and NLRP3 mediate sterile inflammasome activation in microglia and astrocytes. *J. Exp. Med.* 214 1351–1370. 10.1084/jem.20150237 28404595 PMC5413320

[B81] Freund A. Laberge R. M. Demaria M. Campisi J. (2012). Lamin B1 loss is a senescence-associated biomarker. *Mol. Biol. Cell* 23 2066–2075. 10.1091/mbc.E11-10-0884 22496421 PMC3364172

[B82] Fukusumi H. Togo K. Sumida M. Nakamori M. Obika S. Baba K. (2021). Alpha-synuclein dynamics in induced pluripotent stem cell-derived dopaminergic neurons from a Parkinson’s disease patient (PARK4) with SNCA triplication. *FEBS Open Bio* 11 354–366. 10.1002/2211-5463.13060 33301617 PMC7876504

[B83] Galaris D. Barbouti A. Pantopoulos K. (2019). Iron homeostasis and oxidative stress: an intimate relationship. *Biochim. Biophys. Acta Mol. Cell Res.* 1866:118535. 10.1016/j.bbamcr.2019.118535 31446062

[B84] Gao L. Zheng W. G. Wu X. K. Du G. H. Qin X. M. (2021). Baicalein Delays H2O2-Induced Astrocytic Senescence through Inhibition of Senescence-Associated Secretory Phenotype (SASP), Suppression of JAK2/STAT1/NF-κB Pathway, and Regulation of Leucine Metabolism. *ACS Chem. Neurosci.* 12 2320–2335. 10.1021/acschemneuro.1c00024 34152720

[B85] Garcia-Esparcia P. Llorens F. Carmona M. Ferrer I. (2014). Complex deregulation and expression of cytokines and mediators of the immune response in Parkinson’s disease brain is region dependent. *Brain Pathol.* 24 584–598. 10.1111/bpa.12137 24593806 PMC8029304

[B86] Geng L. Fan L. M. Liu F. Smith C. Li J. M. (2020). Nox2 dependent redox-regulation of microglial response to amyloid-β stimulation and microgliosis in aging. *Sci. Rep.* 10 1–11. 10.1038/s41598-020-58422-8 32005915 PMC6994719

[B87] Gerhard A. Pavese N. Hotton G. Turkheimer F. Es M. Hammers A. (2006). In vivo imaging of microglial activation with [11C](R)-PK11195 PET in idiopathic Parkinson’s disease. *Neurobiol. Dis.* 21 404–412. 10.1016/j.nbd.2005.08.002 16182554

[B88] Giasson B. I. Duda J. E. Murray I. V. J. Chen Q. Souza J. M. Hurtig H. I. (2000). Oxidative damage linked to neurodegeneration by selective α-synuclein nitration in synucleinopathy lesions. *Science* 290 985–989. 10.1126/science.290.5493.985 11062131

[B89] Giovannoni F. Quintana F. J. (2020). The role of astrocytes in CNS inflammation. *Trends Immunol.* 41 805–819. 10.1016/j.it.2020.07.007 32800705 PMC8284746

[B90] Goedert M. (1997). The awakening of alpha-synucelin. *Nature* 388 232–233. 10.1111/j.1478-1913.1930.tb00764.x 9230428

[B91] Goedert M. Jakes R. Spillantini M. G. (2017). The Synucleinopathies: twenty Years on. *J. Parkinson’s Dis.* 7 S53–S71. 10.3233/JPD-179005 28282814 PMC5345650

[B92] Gonzales M. M. Garbarino V. R. Marques Zilli E. Petersen R. C. Kirkland J. L. Tchkonia T. (2022). Senolytic Therapy to Modulate the Progression of Alzheimer’s Disease (SToMP-AD): a Pilot Clinical Trial. *J. Prevent. Alzheimer’s Dis.* 9 22–29. 10.14283/jpad.2021.62 35098970 PMC8612719

[B93] González-Barbosa E. Mejía-García A. Bautista E. Gonzalez F. J. Segovia J. Elizondo G. (2017). TCDD induces UbcH7 expression and synphilin-1 protein degradation in the mouse ventral midbrain. *J. Biochem. Mol. Toxicol.* 31 1–8. 10.1002/jbt.21947 28621812 PMC6309283

[B94] Grazia M. Goedert M. (2000). The Alpha-Synucleinopathies: parkinson’s Disease, Dementia with Lewy Bodies, and Multiple. *Mol. Basis Dement.* 920 16–27. 10.1111/j.1749-6632.2000.tb06900.x 11193145

[B95] Gu X. L. Long C. X. Sun L. Xie C. Lin X. Cai H. (2010). Astrocytic expression of Parkinson’s disease-related A53T -synuclein causes neurodegeneration in mice. *Mol. Brain* 3 1–16. 10.1186/1756-6606-3-12 20409326 PMC2873589

[B96] Hald A. Lotharius J. (2005). Oxidative stress and inflammation in Parkinson’s disease: is there a causal link? *Exp. Neurol.* 193 279–290. 10.1016/j.expneurol.2005.01.013 15869932

[B97] Halliday G. Bigio E. H. Cairns N. J. (2012). Mechanisms of disease in frontotemporal lobar degeneration: gain of function versus loss of function effects. *Acta Neuropathol.* 124 373–382. 10.1007/s00401-012-1030-4.Mechanisms 22878865 PMC3445027

[B98] Han X. Zhang T. Liu H. Mi Y. Gou X. (2020). Astrocyte Senescence and Alzheimer’s Disease: a Review. *Front. Aging Neurosci.* 12:1–13. 10.3389/fnagi.2020.00148 32581763 PMC7297132

[B99] Hanamsagar R. Alter M. D. Block C. S. Sullivan H. Bolton J. L. Bilbo S. D. (2017). Generation of a microglial developmental index in mice and in humans reveals a sex difference in maturation and immune reactivity. *Glia* 65 1504–1520. 10.1002/glia.23176 28618077 PMC5540146

[B100] Harman D. (1956). Aging: a theory based on free radical and radiation chemistry. *J. Gerontol.* 11 298–300. 10.1093/geronj/11.3.298 13332224

[B101] Harms A. S. Kordower J. H. Sette A. Lindestam Arlehamn C. S. Sulzer D. Mach R. H. (2021). Inflammation in Experimental Models of α-Synucleinopathies. *Mov. Disord.* 36 37–49. 10.1002/mds.28264 33009855 PMC8115204

[B102] Hartmann A. Mouatt-Prigent A. Vila M. Abbas N. Perier C. Faucheux B. A. (2002). Increased expression and redistribution of the antiapoptotic molecule Bcl-xL in Parkinson’s disease. *Neurobiol. Dis.* 10 28–32. 10.1006/nbdi.2002.0494 12079401

[B103] He S. Zhong S. Liu G. Yang J. (2021). Alpha-Synuclein: the Interplay of Pathology, Neuroinflammation, and Environmental Factors in Parkinson’s Disease. *Neurodegen. Dis.* 20 55–64. 10.1159/000511083 33465773

[B104] Hernández I. H. Villa-González M. Martín G. Soto M. Pérez-álvarez M. J. (2021). Glial cells as therapeutic approaches in brain ischemia-reperfusion injury. *Cells* 10 1–26. 10.3390/cells10071639 34208834 PMC8305833

[B105] Herranz N. Gil J. (2018). Mechanisms and functions of cellular senescence. *J. Clin. Investig.* 128 1238–1246. 10.1172/JCI95148 29608137 PMC5873888

[B106] Hickson L. T. J. Langhi Prata L. G. P. Bobart S. A. Evans T. K. Giorgadze N. Hashmi S. K. (2019). Senolytics decrease senescent cells in humans: preliminary report from a clinical trial of Dasatinib plus Quercetin in individuals with diabetic kidney disease. *EBioMedicine* 47 446–456. 10.1016/j.ebiom.2019.08.069 31542391 PMC6796530

[B107] Hinkle J. T. Dawson V. L. Dawson T. M. (2019). The A1 astrocyte paradigm: new avenues for pharmacological intervention in neurodegeneration. *Mov. Disord.* 34 959–969. 10.1002/mds.27718 31136698 PMC6642014

[B108] Hirsch E. C. Standaert D. G. (2021). Ten Unsolved Questions About Neuroinflammation in Parkinson’s Disease. *Mov. Disord.* 36 16–24. 10.1002/mds.28075 32357266

[B109] Hishikawa N. Hashizume Y. Yoshida M. Sobue G. (2001). Widespread occurrence of argyrophilic glial inclusions in Parkinson’s disease. *Neuropathol. Appl. Neurobiol.* 27 362–372. 10.1046/j.1365-2990.2001.00345.x 11679088

[B110] Hoare M. Narita M. (2013). Transmitting senescence to the cell neighbourhood. *Nat. Cell Biol.* 15 887–889. 10.1038/ncb2811 23907191

[B111] Hoenen C. Gustin A. Birck C. Kirchmeyer M. Beaume N. Felten P. (2016). Alpha-synuclein proteins promote pro-inflammatory cascades in microglia: stronger effects of the a53t mutant. *PLoS One* 11:1–24. 10.1371/journal.pone.0162717 27622765 PMC5021287

[B112] Hsiao C. Bin, Bedi H. Gomez R. Khan A. Meciszewski T. (2021). Telomere length shortening in microglia: implication for accelerated senescence and neurocognitive deficits in HIV. *Vaccines* 9 1–17. 10.3390/vaccines9070721 34358137 PMC8310244

[B113] Ibáñez P. Bonnet A. Débarges B. Lohmann E. Tison F. Pollak P. (2004). Causal relation between alpha-synuclein gene duplication and familial Parkinson’s disease. *Lancet* 364 1169–1171. 10.1016/S0140-6736(04)17104-3 15451225

[B114] Im J. H. Yeo I. J. Jeon S. H. Lee D. H. Ham H. J. Yun J. (2020). *P21 Inhibitor UC2288 Ameliorates MPTP Induced Parkinson’s Disease Progression Through Inhibition of Oxidative Stress and Neuroinflammation.* [Preprint]. 10.21203/rs.3.rs-19130/v1

[B115] Iovino L. Tremblay M. E. Civiero L. (2020). Glutamate-induced excitotoxicity in Parkinson’s disease: the role of glial cells. *J. Pharmacol. Sci.* 144 151–164. 10.1016/j.jphs.2020.07.011 32807662

[B116] Iqbal A. Baldrighi M. Murdoch J. N. Fleming A. Wilkinson C. J. (2020). Alpha-synuclein aggresomes inhibit ciliogenesis and multiple functions of the centrosome. *Biol. Open* 9:54338. 10.1242/bio.054338 32878882 PMC7561473

[B117] Iranzo A. Santamaria J. Tolosa E. (2016). Idiopathic rapid eye movement sleep behaviour disorder: diagnosis, management, and the need for neuroprotective interventions. *Lancet Neurol.* 15 405–419. 10.1016/S1474-4422(16)00057-0 26971662

[B118] Jakes R. Spillantini M. G. Goedert M. (1994). Identification of two distinct synucleins from human brain. *FEBS Lett.* 345 27–32. 10.1016/0014-5793(94)00395-5 8194594

[B119] Jang A. Lee H. J. Suk J. E. Jung J. W. Kim K. P. Lee S. J. (2010). Non-classical exocytosis of α-synuclein is sensitive to folding states and promoted under stress conditions. *J. Neurochem.* 113 1263–1274. 10.1111/j.1471-4159.2010.06695.x 20345754

[B120] Johansen K. K. Torp S. H. Farrer M. J. Gustavsson E. K. Aasly J. O. (2018). A Case of Parkinson’s Disease with No Lewy Body Pathology due to a Homozygous Exon Deletion in Parkin. *Case Rep. Neurol. Med.* 2018 1–4. 10.1155/2018/6838965 30050705 PMC6046180

[B121] Jordán J. Galindo M. F. Tornero D. González-García C. Ceña V. (2004). Bcl-xL blocks mitochondrial multiple conductance channel activation and inhibits 6-OHDA-induced death in SH-SY5Y cells. *J. Neurochem.* 89 124–133. 10.1046/j.1471-4159.2003.02299.x 15030396

[B122] Jung S. Jeong H. Yu S. W. (2020). Autophagy as a decisive process for cell death. *Exp. Mol. Med.* 52 921–930. 10.1038/s12276-020-0455-4 32591647 PMC7338414

[B123] Justice J. N. Nambiar A. M. Tchkonia T. LeBrasseur N. K. Pascual R. Hashmi S. K. (2019). Senolytics in idiopathic pulmonary fibrosis: results from a first-in-human, open-label, pilot study. *EBioMedicine* 40 554–563. 10.1016/j.ebiom.2018.12.052 30616998 PMC6412088

[B124] Kam T. I. Hinkle J. T. Dawson T. M. Dawson V. L. (2020). Microglia and astrocyte dysfunction in parkinson’s disease. *Neurobiol. Dis.* 144:105028. 10.1016/j.nbd.2020.105028 32736085 PMC7484088

[B125] Kanaan N. M. Kordower J. H. Collier T. J. (2008). Age and region-specific responses of microglia, but not astrocytes, suggest a role in selective vulnerability of dopamine neurons after 1-methyl-4-phenyl-1,2,3,6-tetrahydropyridine exposure in monkeys. *Glia* 56 1199–1214. 10.1002/glia.20690 18484101 PMC3388430

[B126] Kang C. Xu Q. Martin T. D. Li M. Z. Demaria M. Aron L. (2015). The DNA damage response induces inflammation and senescence by inhibiting autophagy of GATA4. *Science* 349 1–26. 10.1126/science.aaa5612 26404840 PMC4942138

[B127] Karin O. Alon U. (2021). Senescent cell accumulation mechanisms inferred from parabiosis. *GeroScience* 43 329–341. 10.1007/s11357-020-00286-x 33236264 PMC8050176

[B128] Kellie J. F. Higgs R. E. Ryder J. W. Major A. Beach T. G. Adler C. H. (2014). Quantitative Measurement of Intact Alpha-Synuclein Proteoforms from Post-Mortem Control and Parkinson’s Disease Brain Tissue by Intact Protein Mass Spectrometry. *Sci. Rep.* 4:5797. 10.1038/srep05797 25052239 PMC4107347

[B129] Kennedy B. K. Berger S. L. Brunet A. Campisi J. Cuervo A. M. Epel E. S. (2014). Geroscience: linking aging to chronic disease. *Cell* 159 709–713. 10.1016/j.cell.2014.10.039 25417146 PMC4852871

[B130] Khakh B. S. Sofroniew M. V. (2015). Diversity of astrocyte functions and phenotypes in neural circuits Introduction and historical perspective. *Nat. Neurosci.* 18 942–952. 10.1038/nn.4043.Diversity 26108722 PMC5258184

[B131] Kielb S. Kisanuki Y. Y. Dawson E. (2021). Neuropsychological profile associated with an alpha-synuclein gene (SNCA) duplication. *Clin. Neuropsychol.* 2021 1–12. 10.1080/13854046.2021.1914735 33983072

[B132] Kim C. Ho D. H. Suk J. E. You S. Michael S. Kang J. (2013). Neuron-released oligomeric α-synuclein is an endogenous agonist of TLR2 for paracrine activation of microglia. *Nat. Commun.* 4 1–24. 10.1038/ncomms2534 23463005 PMC4089961

[B133] Kirkland J. L. Tchkonia T. (2020). Senolytic drugs: from discovery to translation. *J. Internal Med.* 288 518–536. 10.1111/joim.13141 32686219 PMC7405395

[B134] Kodama L. Gan L. (2019). Do Microglial Sex Differences Contribute to Sex Differences in Neurodegenerative Diseases? *Trends Mol. Med.* 25 741–749. 10.1016/j.molmed.2019.05.001 31171460 PMC7338035

[B135] Kon T. Tomiyama M. Wakabayashi K. (2020). Neuropathology of Lewy body disease: clinicopathological crosstalk between typical and atypical cases. *Neuropathology* 40 30–39. 10.1111/neup.12597 31498507

[B136] Konishi H. Okamoto T. Hara Y. Komine O. Tamada H. Maeda M. (2020). Astrocytic phagocytosis is a compensatory mechanism for microglial dysfunction. *EMBO J.* 39 1–18. 10.15252/embj.2020104464 32959911 PMC7667883

[B137] Krashia P. Nobili A. D’amelio M. (2019). Unifying hypothesis of dopamine neuron loss in neurodegenerative diseases: focusing on alzheimer’s disease. *Front. Mol. Neurosci.* 12:1–8. 10.3389/fnmol.2019.00123 31156387 PMC6534044

[B138] Kritsilis M. Rizou S. V. Koutsoudaki P. N. Evangelou K. Gorgoulis V. G. Papadopoulos D. (2018). Ageing, cellular senescence and neurodegenerative disease. *Int. J. Mol. Sci.* 19:19102937. 10.3390/ijms19102937 30261683 PMC6213570

[B139] Kumari R. Jat P. (2021). Mechanisms of Cellular Senescence: cell Cycle Arrest and Senescence Associated Secretory Phenotype. *Front. Cell Dev. Biol.* 9:1–24. 10.3389/fcell.2021.645593 33855023 PMC8039141

[B140] Kwon O. Song J. Yang Y. Kim S. Kim J. Y. Seok M. (2021). SGK1 inhibition in glia ameliorates pathologies and symptoms in Parkinson disease animal models. *EMBO Mol. Med.* 13 1–26. 10.15252/emmm.202013076 33646633 PMC8033538

[B141] Lai T. T. Kim Y. J. Nguyen P. T. Koh Y. H. Nguyen T. T. Ma H. (2021). Temporal Evolution of Inflammation and Neurodegeneration With Alpha-Synuclein Propagation in Parkinson’s Disease Mouse Model. *Front. Integrat. Neurosci.* 15:1–17. 10.3389/fnint.2021.715190 34675786 PMC8523784

[B142] Lang F. Voelkl J. (2013). Therapeutic potential of serum and glucocorticoid inducible kinase inhibition. *Expert Opin. Investig. Drugs* 22 701–714. 10.1517/13543784.2013.778971 23506284

[B143] Langhi Prata L. G. P. Ovsyannikova I. G. Tchkonia T. Kirkland J. L. (2018). Senescent cell clearance by the immune system: emerging therapeutic opportunities. *Semin. Immunol.* 40:101275. 10.1016/j.smim.2019.04.003.Senescent PMC7061456 31088710

[B144] Lashuel H. A. (2020). Do Lewy bodies contain alpha-synuclein fibrils? and Does it matter? A brief history and critical analysis of recent reports. *Neurobiol. Dis.* 141:104876. 10.1016/j.nbd.2020.104876 32339655

[B145] Lee H. J. Bae E. J. Lee S. J. (2014). Extracellular alpha-synuclein - a novel and crucial factor in Lew body diseases. *Nat. Rev. Neurol.* 10 92–98. 10.1038/nrneurol.2013.275 24468877

[B146] Lee H. J. Suk J. E. Patrick C. Bae E. J. Cho J. H. Rho S. (2010). Direct transfer of α-synuclein from neuron to astroglia causes inflammatory responses in synucleinopathies. *J. Biol. Chem.* 285 9262–9272. 10.1074/jbc.M109.081125 20071342 PMC2838344

[B147] Lee S. J. Lim H. S. Masliah E. Lee H. J. (2011). Protein aggregate spreading in neurodegenerative diseases: problems and perspectives. *Neurosci. Res.* 70 339–348. 10.1016/j.neures.2011.05.008.Protein 21624403 PMC3912578

[B148] Lee Y. H. Cha J. Chung S. J. Yoo H. S. Sohn Y. H. Ye B. S. (2019). Beneficial effect of estrogen on nigrostriatal dopaminergic neurons in drug-naïve postmenopausal Parkinson’s disease. *Sci. Rep.* 9 1–9. 10.1038/s41598-019-47026-6 31324895 PMC6642214

[B149] Li H. Alavian K. N. Lazrove E. Mehta N. Jones A. Zhang P. (2013). A Bcl-xL -Drp1 complex regulates synaptic vesicle membrane dynamics during endocytosis. *Nat. Cell Biol.* 15 773–785. 10.1038/ncb2791 23792689 PMC3725990

[B150] Li H. Chen Y. Jones A. F. Sanger R. H. Collis L. P. Flannery R. (2008). Bcl-xL induces Drp1-dependent synapse formation in cultured hippocampal neurons. *Proc. Natl. Acad. Sci. USA.* 105 2169–2174. 10.1073/pnas.0711647105 18250306 PMC2542873

[B151] Li L. Hou X. Xu R. Liu C. Tu M. (2017). Research review on the pharmacological effects of astragaloside IV. *Fundament. Clin. Pharmacol.* 31 17–36. 10.1111/fcp.12232 27567103

[B152] Li Q. Barres B. A. (2018). Microglia and macrophages in brain homeostasis and disease. *Nat. Rev. Immunol.* 18 225–242. 10.1038/nri.2017.125 29151590

[B153] Li W. West N. Colla E. Pletnikova O. Troncoso J. C. Marsh L. (2005). Aggregation promoting C-terminal truncation of alpha-synuclein is a normal cellular process and is enhanced by the familial Parkinson’s disease-linked mutations. *Proc. Natl. Acad. Sci. USA.* 102 2162–2167. 10.1073/pnas.0406976102 15684072 PMC548541

[B154] Li Y. Niu M. Zhao A. Kang W. Chen Z. Luo N. (2019). CXCL12 is involved in α-synuclein-triggered neuroinflammation of Parkinson’s disease. *J. Neuroinflamm.* 16 1–14. 10.1186/s12974-019-1646-6 31831012 PMC6909602

[B155] Liddelow S. A. Barres B. A. (2017). Reactive Astrocytes: production, Function, and Therapeutic Potential. *Immunity* 46 957–967. 10.1016/j.immuni.2017.06.006 28636962

[B156] Liddelow S. A. Guttenplan K. A. Clarke L. E. Bennett F. C. Bohlen C. J. Schirmer L. (2017). Neurotoxic reactive astrocytes are induced by activated microglia. *Nature* 541 481–487. 10.1038/nature21029 28099414 PMC5404890

[B157] Lim J. Kim M. J. Park Y. J. Ahn J. W. Hwang S. J. Moon J. S. (2019). Upregulation of the NLRC4 inflammasome contributes to poor prognosis in glioma patients. *Sci. Rep.* 9 1–10. 10.1038/s41598-019-44261-9 31133717 PMC6536517

[B158] Limbad C. Oron T. R. Alimirah F. Davalos A. R. Tracy T. E. Gan L. (2020). Astrocyte senescence promotes glutamate toxicity in cortical neurons. *PLoS One* 15:1–17. 10.1371/journal.pone.0227887 31945125 PMC6964973

[B159] Liu C. Giasson B. I. Lewis K. A. Lee V. M. Demartino G. N. Thomas P. J. (2005). A Precipitating Role for Truncated Alpha-Synuclein and the Proteasome in Alpha-Synuclein Aggregation. *J. Biol. Chem.* 280 22670–22678. 10.1074/jbc.M501508200 15840579

[B160] Liu G. Hosomi N. Hitomi H. Pelisch N. Fu H. Masugata H. (2011). Angiotensin II induces human astrocyte senescence through reactive oxygen species production. *Hypertens. Res.* 34 479–483. 10.1038/hr.2010.269 21270817

[B161] Liu J. Liu W. Li R. Yang H. (2019). Mitophagy in Parkinson’s Disease: from Pathogenesis to Treatment. *Cells* 8:712. 10.3390/cells8070712 31336937 PMC6678174

[B162] Liu L. R. Liu J. C. Bao J. S. Bai Q. Q. Wang G. Q. (2020). Interaction of Microglia and Astrocytes in the Neurovascular Unit. *Front. Immunol.* 11:1–11. 10.3389/fimmu.2020.01024 32733433 PMC7362712

[B163] Liu T. Zhang L. Joo D. Sun S. C. (2017). NF-κB signaling in inflammation. *Signal Transduc. Targeted Therapy* 2:23. 10.1038/sigtrans.2017.23 29158945 PMC5661633

[B164] Lopes K. O. Sparks D. L. Streit W. J. (2008). Microglial dystrophy in the aged and Alzheimer’s disease brain is associated with ferritin immunoreactivity. *Glia* 56 1048–1060. 10.1002/glia.20678 18442088

[B165] López-Otín C. Blasco M. A. Partridge L. Serrano M. Kroemer G. (2013). The hallmarks of aging. *Cell* 153:1194. 10.1016/j.cell.2013.05.039 23746838 PMC3836174

[B166] Loria F. Vargas J. Y. Bousset L. Syan S. Salles A. Melki R. (2017). α-Synuclein transfer between neurons and astrocytes indicates that astrocytes play a role in degradation rather than in spreading. *Acta Neuropathol.* 134 789–808. 10.1007/s00401-017-1746-2 28725967

[B167] Lull M. E. Block M. L. (2010). Microglial Activation and Chronic Neurodegeneration. *Neurotherapeutics* 7 354–365. 10.1016/j.nurt.2010.05.014 20880500 PMC2951017

[B168] Luo X. Ding J. Chen S. (2010). Microglia in the aging brain: relevance to neurodegeneration. *Mol. Neurodegen.* 5 1–9. 10.1186/1750-1326-5-12 20334662 PMC2852379

[B169] Ma L. Yang C. Zhang X. Li Y. Wang S. Zheng L. (2018). C-terminal truncation exacerbates the aggregation and cytotoxicity of α-Synuclein: a vicious cycle in Parkinson’s disease. *BBA Mol. Basis Dis.* 1864 3714–3725. 10.1016/j.bbadis.2018.10.003 30290273

[B170] Maciel-Barón L. A. Morales-Rosales S. L. Aquino-Cruz A. A. Triana-Martínez F. Galván-Arzate S. Luna-López A. (2016). Senescence associated secretory phenotype profile from primary lung mice fibroblasts depends on the senescence induction stimuli. *Age* 38 1–14. 10.1007/s11357-016-9886-1 26867806 PMC5005892

[B171] Mahul-Mellier A. L. Burtscher J. Maharjan N. Weerens L. Croisier M. Kuttler F. (2020). The process of Lewy body formation, rather than simply α-synuclein fibrillization, is one of the major drivers of neurodegeneration. *Proc. Natl. Acad. Sci. USA.* 117 4971–4982. 10.1073/pnas.1913904117 32075919 PMC7060668

[B172] Marras C. Canning C. G. Goldman S. M. (2019). Environment, lifestyle, and Parkinson’s disease: implications for prevention in the next decade. *Mov. Disord.* 34 801–811. 10.1002/mds.27720 31091353

[B173] Martínez-Cué C. Rueda N. (2020). Cellular Senescence in Neurodegenerative Diseases. *Front. Cell. Neurosci.* 14:16. 10.3389/fncel.2020.00016 32116562 PMC7026683

[B174] Mas-Bargues C. Borrás C. Viña J. (2021). Bcl-xl as a modulator of senescence and aging. *Int. J. Mol. Sci.* 22 1–15. 10.3390/ijms22041527 33546395 PMC7913597

[B175] Masuda T. Sankowski R. Staszewski O. Prinz M. (2020). Microglia Heterogeneity in the Single-Cell Era. *Cell Rep.* 30 1271–1281. 10.1016/j.celrep.2020.01.010 32023447

[B176] Matejuk A. Ransohoff R. M. (2020). Crosstalk Between Astrocytes and Microglia: an Overview. *Front. Immunol.* 11:1–11. 10.3389/fimmu.2020.01416 32765501 PMC7378357

[B177] Mavroeidi P. Xilouri M. (2021). Neurons and glia interplay in α-synucleinopathies. *Int. J. Mol. Sci.* 22:22094994. 10.3390/ijms22094994 34066733 PMC8125822

[B178] McCann H. Stevens C. H. Cartwright H. Halliday G. M. (2014). α-Synucleinopathy phenotypes. *Parkinson. Related Disord.* 20(Suppl. 1), S62–S67. 10.1016/S1353-8020(13)70017-8 24262191

[B179] McConnell B. B. Starborg M. Brookes S. Peters G. (1998). Inhibitors of cyclin-dependent kinases induce features of replicative senescence in early passage human diploid fibroblasts. *Curr. Biol.* 8 351–354. 10.1016/S0960-9822(98)70137-X 9512419

[B180] McCormack A. L. Mak S. K. Di Monte D. A. (2012). Increased a-synuclein phosphorylation and nitration in the aging primate substantia nigra. *Cell Death Dis.* 3 1–9. 10.1038/cddis.2012.50 22647852 PMC3366084

[B181] Miller D. W. Hague S. M. Clarimon J. Baptista M. Gwinn-Hardy K. Cookson M. R. (2004). Alpha-Synuclein in blood and brain from familial Parkinson disease with SNCA locus triplication. *Neurology* 62 1835–1838. 10.1212/01.wnl.0000127517.33208.f4 15159488

[B182] Miller S. J. (2018). Astrocyte heterogeneity in the adult central nervous system. *Front. Cell. Neurosci.* 12:1–6. 10.3389/fncel.2018.00401 30524236 PMC6262303

[B183] Mittal S. Bjørnevik K. Im D. S. Flierl A. Dong X. Locascio J. J. (2017). β2-Adrenoreceptor is a regulator of the α-synuclein gene driving risk of Parkinson’s disease. *Science* 357 891–898. 10.1126/science.aaf3934 28860381 PMC5761666

[B184] Mitteldorf J. (2019). What is Antagonistic Pleiotropy? *Biochemistry* 84 1458–1468. 10.1101/321588 31870250

[B185] Moreno-blas D. Gorostieta-salas E. Pommer-alba A. Muciño-G. Gerónimo-olvera C. Maciel-barón L. A. (2019). Cortical neurons develop a senescence-like phenotype promoted by dysfunctional autophagy. *Aging* 11 6175–6198. 10.18632/aging.102181 31469660 PMC6738425

[B186] Nagata S. Tanaka M. (2017). Programmed cell death and the immune system. *Nat. Rev. Immunol.* 17 333–340. 10.1038/nri.2016.153 28163302

[B187] Nakamura K. Oshima T. Morimoto T. Ikeda S. Yoshikawa H. Shiwa Y. (2011). Sequence-specific error profile of Illumina sequencers. *Nucleic Acids Res.* 39:gkr344. 10.1093/nar/gkr344 21576222 PMC3141275

[B188] Nalls M. A. Blauwendraat C. Vallerga C. L. Heilbron K. Bandres-Ciga S. Chang D. (2019). Identification of novel risk loci, causal insights, and heritable risk for Parkinson’s disease: a meta-analysis of genome-wide association studies. *Lancet Neurol.* 18 1091–1102. 10.1016/S1474-4422(19)30320-5 31701892 PMC8422160

[B189] Nelson G. Wordsworth J. Wang C. Jurk D. Lawless C. Martin-Ruiz C. (2012). A senescent cell bystander effect: senescence-induced senescence. *Aging Cell* 11 345–349. 10.1111/j.1474-9726.2012.00795.x 22321662 PMC3488292

[B190] Norden D. M. Godbout J. P. (2013). Microglia of the aged brain: primed to be activated and resistant to regulation. *Neuropathol. Appl. Neurobiol.* 39 19–34. 10.1111/j.1365-2990.2012.01306.x.Microglia 23039106 PMC3553257

[B191] Olah M. Biber K. Vinet J. Boddeke W. G. M. (2011). Microglia Phenotype Diversity. *CNS Neurol. Disord. Drug Targets* 10 108–118. 10.2174/187152711794488575 21143141

[B192] Olgiati S. Thomas A. Quadri M. Breedveld G. J. Graafland J. Eussen H. (2015). Early-onset parkinsonism caused by alpha-synuclein gene triplication: clinical and genetic fi ndings in a novel family. *Parkins. Related Disord.* 21 981–986. 10.1016/j.parkreldis.2015.06.005 26077166

[B193] Ono K. (2017). The Oligomer Hypothesis in α-Synucleinopathy. *Neurochem. Res.* 42 3362–3371. 10.1007/s11064-017-2382-x 28828740

[B194] Osman A. M. Sun Y. Burns T. C. He L. Kee N. Oliva-Vilarnau N. (2020). Radiation Triggers a Dynamic Sequence of Transient Microglial Alterations in Juvenile Brain. *Cell Rep.* 31 107699. 10.1016/j.celrep.2020.107699 32492415

[B195] Ouchi Y. (2017). Imaging neuroinflammation to monitor α-synucleinopathy. *Lancet Neurol.* 16 763–764. 10.1016/S1474-4422(17)30244-2 28684246

[B196] Ovadya Y. Krizhanovsky V. (2018). Strategies targeting cellular senescence. *J. Clin. Investig.* 128 1247–1254. 10.1172/JCI95149 29608140 PMC5873866

[B197] Özcan S. Alessio N. Acar M. B. Mert E. Omerli F. Peluso G. (2016). Unbiased analysis of senescence associated secretory phenotype (SASP) to identify common components following different genotoxic stresses. *Aging* 8 1316–1329. 10.18632/aging.100971 27288264 PMC4993333

[B198] Park H. A. Jonas E. A. (2017). Δn-Bcl-xL, a therapeutic target for neuroprotection. *Neural Regen. Res.* 12 1791–1794. 10.4103/1673-5374.219033 29239317 PMC5745825

[B199] Park H. A. Licznerski P. Alavian K. N. Shanabrough M. Jonas E. A. (2015). Bcl-xL is necessary for neurite outgrowth in hippocampal neurons. *Antioxid. Redox Signal.* 22 93–108. 10.1089/ars.2013.5570 24787232 PMC4281845

[B200] Park H. A. Licznerski P. Mnatsakanyan N. Niu Y. Sacchetti S. Wu J. (2017). Inhibition of Bcl-xL prevents pro-death actions of ΔN-Bcl-xL at the mitochondrial inner membrane during glutamate excitotoxicity. *Cell Death Different.* 24 1963–1974. 10.1038/cdd.2017.123 28777375 PMC5635221

[B201] Peng C. Trojanowski J. Q. Lee V. M. Y. (2020). Protein transmission in neurodegenerative disease. *Nat. Rev. Neurol.* 16 199–212. 10.1038/s41582-020-0333-7 32203399 PMC9242841

[B202] Phinney A. L. Andringa G. Bol J. G. J. M. Wolters E. C. Muiswinkel F. L. Dam A. M. W. (2006). Enhanced sensitivity of dopaminergic neurons to rotenone-induced toxicity with aging. *Parkins. Related Disord.* 12 228–238. 10.1016/j.parkreldis.2005.12.002 16488175

[B203] Polymeropoulos M. H. Lavedan C. Leroy E. Ide S. E. Dehejia A. Dutra A. (1997). Mutation in the α-synuclein gene identified in families with Parkinson’s disease. *Science* 276 2045–2047. 10.1126/science.276.5321.2045 9197268

[B204] Puschmann A. Ross O. A. Vilariño-Güell C. Lincoln S. J. Kachergus J. M. Cobb S. A. (2009). A Swedish family with de novo α-synuclein A53T mutation: evidence for early cortical dysfunction. *Parkins. Relat. Disord.* 15 627–632. 10.1016/j.parkreldis.2009.06.007.A PMC2783246 19632874

[B205] Rannikko E. H. Weber S. S. Kahle P. J. (2015). Exogenous α-synuclein induces toll-like receptor 4 dependent inflammatory responses in astrocytes. *BMC Neurosci.* 16:1–11. 10.1186/s12868-015-0192-0 26346361 PMC4562100

[B206] Reeve A. Simcox E. Turnbull D. (2014). Ageing and Parkinson’s disease: why is advancing age the biggest risk factor? *Ageing Res. Rev.* 14 19–30. 10.1016/j.arr.2014.01.004 24503004 PMC3989046

[B207] Rezaie P. Cairns N. J. Chadwick A. Lantos P. L. (1996). Lewy bodies are located preferentially in limbic areas in diffuse Lewy body disease. *Neurosci. Lett.* 212 111–114. 10.1016/0304-3940(96)12775-0 8832651

[B208] Riessland M. (2020). Is Cellular Senescence of Dopaminergic Neurons the Cause of Local Inflammation in the Midbrain Observed in Parkinson’s Disease? *J. Cell. Immunol.* 2:43. 10.33696/immunology.2.043

[B209] Riessland M. Kolisnyk B. Kim T. W. Cheng J. Ni J. Pearson J. A. (2019). Loss of SATB1 Induces p21-Dependent Cellular Senescence in Post-mitotic Dopaminergic Neurons. *Cell Stem Cell* 25 514.e–530.e. 10.1016/j.stem.2019.08.013 31543366 PMC7493192

[B210] Ritzel R. M. Doran S. J. Glaser E. P. Meadows V. E. Faden A. L. Stoica A. S. (2019). Old age increases microglial senescence, exacerbates secondary neuroinflammation, and worsens neurological outcomes following acute traumatic brain injury in mice. *Neurobiol. Aging* 77 194–206. 10.1016/j.neurobiolaging.2019.02.010 30904769 PMC6486858

[B211] Rodriguez M. Rodriguez-Sabate C. Morales I. Sanchez A. Sabate M. (2015). Parkinson’s disease as a result of aging. *Aging Cell* 14 293–308. 10.1111/acel.12312 25677794 PMC4406659

[B212] Romashkan S. Chang H. Hadley E. C. (2021). National institute on aging workshop: repurposing drugs or dietary supplements for their senolytic or senomorphic effects: considerations for clinical trials. *J. Gerontol.* 76 1144–1152. 10.1093/gerona/glab028 33528569 PMC8521777

[B213] Roodveldt C. Labrador-Garrido A. Gonzalez-Rey E. Fernandez-Montesinos R. Caro M. Lachaud C. C. (2010). Glial innate immunity generated by non-aggregated alpha-synuclein in mouse: differences between wild-type and Parkinson’s disease-linked mutants. *PLoS One* 5:13481. 10.1371/journal.pone.0013481 21048992 PMC2964342

[B214] Rostami J. Holmqvist S. Lindström V. Sigvardson J. Westermark G. T. Ingelsson M. (2017). Human astrocytes transfer aggregated alpha-synuclein via tunneling nanotubes. *J. Neurosci.* 37 11835–11853. 10.1523/JNEUROSCI.0983-17.2017 29089438 PMC5719970

[B215] Saez-Atienzar S. Bonet-Ponce L. da Casa C. Perez-Dolz L. Blesa J. R. Nava E. (2016). Bcl-xL-mediated antioxidant function abrogates the disruption of mitochondrial dynamics induced by LRRK2 inhibition. *Biochim. Biophys. Acta Mol. Basis Dis.* 1862 20–31. 10.1016/j.bbadis.2015.09.021 26435084

[B216] Saito Y. Roberu N. N. Sawabe M. Aral T. Kazama H. Hosoi T. (2004). Lewy Body-Related Alpha-Synucleinopathy in Aging. *J. Neuropathol. Exp. Neurol.* 63 742–749. 10.1093/jnen/63.7.742 15290899

[B217] Salas I. H. Burgado J. Allen N. J. (2020). Glia: victims or villains of the aging brain? *Neurobiol. Dis.* 143:105008. 10.1016/j.nbd.2020.105008 32622920

[B218] Sawada H. Hishida R. Hirata Y. Ono K. Suzuki H. Muramatsu S. (2007). Activated Microglia Affect the Nigro- Striatal Dopamine Neurons Differently in Neonatal and Aged Mice Treated with 1-Methyl-4-Phenyl-1,2,3,6- Tetrahydropyridine. *J. Neurosci. Res.* 85 1752–1761. 10.1002/jnr.21241 17469135

[B219] Schaser A. J. Osterberg V. R. Dent S. E. Stackhouse T. L. Wakeham C. M. Boutros S. W. (2019). Alpha-synuclein is a DNA binding protein that modulates DNA repair with implications for Lewy body disorders. *Sci. Rep.* 9 1–19. 10.1038/s41598-019-47227-z 31358782 PMC6662836

[B220] Schiess M. C. Barnes J. L. Ellmore T. M. Poindexter B. J. Dinh K. Bick R. J. (2010). CSF from Parkinson disease Patients Differentially Affects Cultured Microglia and Astrocytes. *BMC Neurosci.* 11:151. 10.1186/1471-2202-11-151 21114836 PMC3012671

[B221] Shaerzadeh F. Phan L. Miller D. Dacquel M. Hachmeister W. Hansen C. (2020). Microglia senescence occurs in both substantia nigra and ventral tegmental area. *Glia* 68 2228–2245. 10.1002/glia.23834 32275335 PMC8356201

[B222] Shahidehpour R. K. Higdon R. E. Crawford N. G. Neltner J. H. Ighodaro E. T. Patel E. (2021). Dystrophic microglia are associated with neurodegenerative disease and not healthy aging in the human brain. *Neurobiol. Aging* 99 19–27. 10.1016/j.neurobiolaging.2020.12.003 33422891 PMC8293930

[B223] Sheeler C. Rosa J. G. Ferro A. McAdams B. Borgenheimer E. Cvetanovic M. (2020). Glia in neurodegeneration: the housekeeper, the defender and the perpetrator. *Int. J. Mol. Sci.* 21 1–16. 10.3390/ijms21239188 33276471 PMC7730416

[B224] Si Z. Sun L. Wang X. (2021). Evidence and perspectives of cell senescence in neurodegenerative diseases. *Biomed. Pharmacother.* 137:111327. 10.1016/j.biopha.2021.111327 33545662

[B225] Siani F. Greco R. Levandis G. Ghezzi C. Daviddi F. Demartini C. (2017). Influence of Estrogen Modulation on Glia Activation in a Murine Model of Parkinson’s Disease. *Front. Neurosci.* 11:1–11. 10.3389/fnins.2017.00306 28620274 PMC5449471

[B226] Sierra A. Gottfried-Blackmore A. C. McEwen B. S. Bulloch K. (2007). Microglia Derived from Aging Mice Exhibit an Altered Inflammatory Profile. *Glia* 55 412–424. 10.1002/glia.20468 17203473

[B227] Sikora E. Bielak-Zmijewska A. Dudkowska M. Krzystyniak A. Mosieniak G. Wesierska M. (2021). Cellular Senescence in Brain Aging. *Front. Aging Neurosci.* 13:1–23. 10.3389/fnagi.2021.646924 33732142 PMC7959760

[B228] Simmnacher K. Krach F. Schneider Y. Alecu J. E. Mautner L. Klein P. (2020). Unique signatures of stress-induced senescent human astrocytes. *Exp. Neurol.* 334:113466. 10.1016/j.expneurol.2020.113466 32949572

[B229] Singleton A. B. Farrer M. Johnson J. Singleton A. Hague S. Kachergus J. (2003). Alpha-Synuclein Locus Triplication Causes Parkinson’s Disease. *Science* 302:841. 10.1126/science.1090278 14593171

[B230] Singleton A. Gwinn-Hardy K. (2004). Parkinson’s disease and dementia with Lewy bodies: a difference in dose? *Lancet* 364 1105–1107. 10.1016/S0140-6736(04)17117-1 15451205

[B231] Smith W. W. Jiang H. Pei Z. Tanaka Y. Morita H. Sawa A. (2005). Endoplasmic reticulum stress and mitochondrial cell death pathways mediate A53T mutant alpha-synuclein-induced toxicity. *Hum. Mol. Genet.* 14 3801–3811. 10.1093/hmg/ddi396 16239241

[B232] Song S. Tchkonia T. Jiang J. Kirkland J. L. Sun Y. (2020). Targeting Senescent Cells for a Healthier Aging: challenges and Opportunities. *Adv. Sci.* 7 1–14. 10.1002/advs.202002611 33304768 PMC7709980

[B233] Soto-Gamez A. Demaria M. (2017). Therapeutic interventions for aging: the case of cellular senescence. *Drug Discov. Today* 22 786–795. 10.1016/j.drudis.2017.01.004 28111332

[B234] Spillantini M. G. Goedert M. (2000). The Alpha-Synucleinopathies: parkinson’s Disease, Dementia with Lewy Bodies, and Multiple System Atrophy. *Ann. N Y. Acad. Sci.* 920 16–27. 11193145 10.1111/j.1749-6632.2000.tb06900.x

[B235] Spillantini M. G. Goedert M. (2013). Tau pathology and neurodegeneration. *Lancet Neurol.* 12 609–622. 10.1016/S1474-4422(13)70090-5 23684085

[B236] Stefanis L. (2012). α-Synuclein in Parkinson’s disease. *Cold Spring Harb. Perspect. Med.* 2 1–23. 10.1101/cshperspect.a009399 22355802 PMC3281589

[B237] Storer M. Keyes W. M. (2014). Developing senescence to remodel the embryo. *Communicat. Integrat. Biol.* 7:29098. 10.4161/cib.29098 26842300 PMC4594451

[B238] Streit W. J. Braak H. Xue Q. S. Bechmann I. (2009). Dystrophic (senescent) rather than activated microglial cells are associated with tau pathology and likely precede neurodegeneration in Alzheimer’s disease. *Acta Neuropathol.* 118 475–485. 10.1007/s00401-009-0556-6 19513731 PMC2737117

[B239] Streit W. J. Sammons N. W. Kuhns A. J. Sparks D. L. (2004). Dystrophic Microglia in the Aging Human Brain. *Glia* 45 208–212. 10.1002/glia.10319 14730714

[B240] Streit W. J. Walter S. A. Pennell N. A. (1999). Reactive microgliosis. *Progress Neurobiol.* 57 563–581. 10.1016/S0301-0082(98)00069-0 10221782

[B241] Streit W. J. Xue Q. S. Tischer J. Bechmann I. (2014). Microglial pathology. *Acta Neuropathol. Commun.* 2 1–17. 10.1186/s40478-014-0142-6 25257319 PMC4180960

[B242] Su X. Maguire-Zeiss K. A. Giuliano R. Prifti L. Venkatesh K. Federoff H. J. (2008). Synuclein activates microglia in a model of Parkinson’s Disease. *Neurobiol. Aging* 29 1690–1701. 10.1016/j.neurobiolaging.2007.04.006.Synuclein 17537546 PMC2621109

[B243] Su Y. Chen Z. Du H. Liu R. Wang W. Li H. (2019). Silencing miR-21 induces polarization of astrocytes to the A2 phenotype and improves the formation of synapses by targeting glypican 6 via the signal transducer and activator of transcription-3 pathway after acute ischemic spinal cord injury. *FASEB J.* 33 10859–10871. 10.1096/fj.201900743R 31266356

[B244] Sugama S. Yang L. Cho B. P. DeGiorgio L. A. Lorenzl S. Albers D. S. (2003). Age-related microglial activation in 1-methyl-4-phenyl-1,2,3,6- tetrahydropyridine (MPTP)-induced dopaminergic neurodegeneration in C57BL/6 mice. *Brain Res.* 964 288–294. 10.1016/S0006-8993(02)04085-4 12576189

[B245] Suzuki M. Sango K. Wada K. Nagai Y. (2018). Pathological role of lipid interaction with α-synuclein in Parkinson’s disease. *Neurochem. Int.* 119 97–106. 10.1016/j.neuint.2017.12.014 29305919

[B246] Tabrizi S. J. Orth M. Max Wilkinson J. Taanman J. W. Warner T. T. Mark Cooper J. (2000). Expression of mutant α-synuclein causes increased susceptibility to dopamine toxicity. *Hum. Mol. Genet.* 9 2683–2689. 10.1093/hmg/9.18.2683 11063727

[B247] Tan Y. L. Yuan Y. Tian L. (2020). Microglial regional heterogeneity and its role in the brain. *Mol. Psychiat.* 25 351–367. 10.1038/s41380-019-0609-8 31772305 PMC6974435

[B248] Tanaka Y. Engelender S. Igarashi S. Rao R. K. Wanner T. Tanzi R. E. (2001). Inducible expression of mutant α-synuclein decreases proteasome activity and increases sensitivity to mitochondria-dependent apoptosis. *Hum. Mol. Genet.* 10 919–926. 10.1093/hmg/10.9.919 11309365

[B249] Tapias V. Hu X. Luk K. C. Sander L. H. Lee V. M. Greenamyre J. T. (2017). Synthetic alpha-synuclein fibrils cause mitochondrial impairment and selective dopamine neurodegeneration in part via iNOS-mediated nitric oxide production. *Cell Mol. Life Sci.* 74 2851–2874. 10.1007/s00018-017-2541-x.SYNTHETIC 28534083 PMC5524146

[B250] Tchieu J. Calder E. L. Guttikonda S. R. Gutzwiller E. M. Aromolaran K. A. Steinbeck J. A. (2019). NFIA is a gliogenic switch enabling rapid derivation of functional human astrocytes from pluripotent stem cells. *Nat. Biotechnol.* 37 267–275. 10.1038/s41587-019-0035-0 30804533 PMC6591152

[B251] Tchkonia T. Palmer A. K. Kirkland J. L. (2021). New Horizons: novel Approaches to Enhance Healthspan through Targeting Cellular Senescence and Related Aging Mechanisms. *J. Clin. Endocrinol. Metabol.* 106 E1481–E1487. 10.1210/clinem/dgaa728 33155651 PMC7947756

[B252] Theillet F. X. Binolfi A. Bekei B. Martorana A. Rose H. M. Stuiver M. (2016). Structural disorder of monomeric α-synuclein persists in mammalian cells. *Nature* 530 45–50. 10.1038/nature16531 26808899

[B253] Touchman J. W. Dehejia A. Chiba-Falek O. Cabin D. E. Schwartz J. R. Orrison B. M. (2001). Human and mouse α-synuclein genes: comparative genomic sequence analysis and identification of a novel gene regulatory element. *Genome Res.* 11 78–86. 10.1101/gr.165801 11156617 PMC311023

[B254] Troncoso-Escudero P. Parra A. Nassif M. Vidal R. L. (2018). Outside in: unraveling the role of neuroinflammation in the progression of Parkinson’s disease. *Front. Neurol.* 9:1–15. 10.3389/fneur.2018.00860 30459700 PMC6232883

[B255] Tsunemi T. Ishiguro Y. Yoroisaka A. Valdez C. Miyamoto K. Ishikawa K. (2020). Astrocytes protect human dopaminergic neurons from α-synuclein accumulation and propagation. *J. Neurosci.* 40 8618–8628. 10.1523/JNEUROSCI.0954-20.2020 33046546 PMC7643299

[B256] Ulusoy A. Febbraro F. Jensen P. H. Kirik D. Romero-Ramos M. (2010). Co-expression of C-terminal truncated alpha-synuclein enhances full-length alpha-synuclein-induced pathology. *Eur. J. Neurosci.* 32 409–422. 10.1111/j.1460-9568.2010.07284.x 20704592

[B257] Uversky V. N. (2003). A protein-chameleon: conformational plasticity of α-synuclein, a disordered protein involved in neurodegenerative disorders. *J. Biomol. Struct. Dynam.* 21 211–234. 10.1080/07391102.2003.10506918 12956606

[B258] van Leeuwen E. Hampton M. B. Smyth L. C. D. (2020). Redox signalling and regulation of the blood-brain barrier. *Int. J. Biochem. Cell Biol.* 125:105794. 10.1016/j.biocel.2020.105794 32562769

[B259] Vasileiou P. Evangelou K. Vlasis K. Fildisis G. Panayiotidis M. Chronopoulos E. (2019). Mitochondrial Homeostasis and Cellular Senescence. *Cells* 8:686. 10.3390/cells8070686 31284597 PMC6678662

[B260] Verkhratsky A. Nedergaard M. (2018). Physiology of astroglia. *Physiol. Rev.* 98 239–389. 10.1152/physrev.00042.2016 29351512 PMC6050349

[B261] Verma D. K. Seo B. A. Ghosh A. Ma S. Hernandez-quijada K. Andersen J. K. (2021). Alpha-Synuclein Preformed Fibrils Induce Cellular Senescence in Parkinson’s Disease Models. *Cells* 10 1–21. 10.3390/cells10071694 34359864 PMC8304385

[B262] Vicencio J. M. Galluzzi L. Tajeddine N. Ortiz C. Criollo A. Tasdemir E. (2008). Senescence, apoptosis or autophagy? When a damaged cell must decide its path - A mini-review. *Gerontology* 54 92–99. 10.1159/000129697 18451641

[B263] Vieira B. D. M. Radford R. A. W. Hayashi J. Eaton E. D. Greenaway B. Jambas M. (2020). Extracellular alpha-synuclein promotes a neuroinhibitory secretory phenotype in astrocytes. *Life* 10 1–14. 10.3390/life10090183 32911644 PMC7555668

[B264] Villa A. Gelosa P. Castiglioni L. Cimino M. Rizzi N. Pepe G. (2018). Sex-Specific Features of Microglia from Adult Mice. *Cell Rep.* 23 3501–3511. 10.1016/j.celrep.2018.05.048 29924994 PMC6024879

[B265] Vinueza-Gavilanes R. Íñigo-Marco I. Larrea L. Lasa M. Carte B. Santamaría E. (2020). N-terminal acetylation mutants affect alpha-synuclein stability, protein levels and neuronal toxicity. *Neurobiol. Dis.* 137:104781. 10.1016/j.nbd.2020.104781 31991248

[B266] Volpicelli-Daley L. A. Gamble K. L. Schultheiss C. E. Riddle D. M. West A. B. Lee V. M. Y. (2014). Formation of α-synuclein lewy neurite-like aggregates in axons impedes the transport of distinct endosomes. *Mol. Biol. Cell* 25 4010–4023. 10.1091/mbc.E14-02-0741 25298402 PMC4263445

[B267] von Bernhardi R. Eugenín-von Bernhardi L. Eugenín J. (2015). Microglial cell dysregulation in brain aging and neurodegeneration. *Front. Aging Neurosci.* 7:1–21. 10.3389/fnagi.2015.00124 26257642 PMC4507468

[B268] Wakabayashi K. (2020). Where and how alpha-synuclein pathology spreads in Parkinson’s disease. *Neuropathology* 40 415–425. 10.1111/neup.12691 32750743

[B269] Wakabayashi K. Hayashi S. Yoshimoto M. Kudo Y. H. Takahashi H. (2000). NACP alphasynuclein-positive filamentous inclusions. *Acta Neuropathol.* 99 14–20. 10.1007/pl00007400 10651022

[B270] Wan C. Liu J. Nie X. Zhao J. Zhou S. Duan Z. (2014). 2,3,7,8-Tetrachlorodibenzo-P-Dioxin (TCDD) induces premature senescence in human and rodent neuronal cells via ROS-dependent mechanisms. *PLoS One* 9:1–10. 10.1371/journal.pone.0089811 24587053 PMC3933666

[B271] Wang Q. Ren N. Cai Z. Lin Q. Wang Z. Zhang Q. (2017). Paraquat and MPTP induce neurodegeneration and alteration in the expression profile of microRNAs: the role of transcription factor Nrf2. *NPJ Parkins. Dis.* 3:1. 10.1038/s41531-017-0033-1 29071302 PMC5651826

[B272] Wang S. Chu C. H. Stewart T. Ginghina C. Wang Y. Nie H. (2015). α-Synuclein, a chemoattractant, directs microglial migration via H2O2-dependent Lyn phosphorylation. *Proc. Natl. Acad. Sci. USA.* 112 E1926–E1935. 10.1073/pnas.1417883112 25825709 PMC4403145

[B273] Wang W. Nguyen L. T. T. Burlak C. Chegini F. Guo F. Chataway T. (2016). Caspase-1 causes truncation and aggregation of the Parkinson’s disease-associated protein α-synuclein. *Proc. Natl. Acad. Sci. USA.* 113 9587–9592. 10.1073/pnas.1610099113 27482083 PMC5003239

[B274] Wang Y. Shi M. Chung K. A. Zabetian C. P. Leverenz J. B. Berg D. (2012). Phosphorylated α-Synuclein in Parkinson’s Disease. *Sci. Translat. Med.* 4 ra20–ra121. 10.1126/scitranslmed.3002566 22344688 PMC3302662

[B275] Wasserman J. K. Schlichter L. C. (2008). White matter injury in young and aged rats after intracerebral hemorrhage. *Exp. Neurol.* 214 266–275. 10.1016/j.expneurol.2008.08.010 18848934

[B276] Watson M. B. Richter F. Lee S. K. Gabby L. Wu J. Masliah E. (2012). Regionally-specific microglial activation in young mice over- expressing human wildtype alpha-synuclein. *Exp. Neurol.* 237 318–334. 10.1016/j.expneurol.2012.06.025 22750327 PMC3443323

[B277] Weed D. L. (2021). Does paraquat cause Parkinson’s disease? A review of reviews. *NeuroToxicology* 86 180–184. 10.1016/j.neuro.2021.08.006 34400206

[B278] Wissler Gerdes E. O. Zhu Y. Tchkonia T. Kirkland J. L. (2020). Discovery, development, and future application of senolytics: theories and predictions. *FEBS J.* 287 2418–2427. 10.1111/febs.15264 32112672 PMC7302972

[B279] Wong W. T. (2013). Microglial aging in the healthy CNS: phenotypes, drivers, and rejuvenation. *Front. Cell. Neurosci.* 7:1–13. 10.3389/fncel.2013.00022 23493481 PMC3595516

[B280] Woodburn S. C. Bollinger J. L. Wohleb E. S. (2021). The semantics of microglia activation: neuroinflammation, homeostasis, and stress. *J. Neuroinflamm.* 18 1–16. 10.1186/s12974-021-02309-6 34742308 PMC8571840

[B281] Wooten G. F. Currie L. J. Bovbjerg V. E. Lee J. K. Patrie J. (2004). Are men at greater risk for Parkinson’s disease than women? *J. Neurol. Neurosurg. Psychiat.* 75 637–639. 10.1136/jnnp.2003.020982 15026515 PMC1739032

[B282] Xia M. L. Xie X. H. Ding J. H. Du R. H. Hu G. (2020). Astragaloside IV inhibits astrocyte senescence: implication in Parkinson’s disease. *J. Neuroinflamm.* 17 1–13. 10.1186/s12974-020-01791-8 32252767 PMC7137443

[B283] Ye Q. Wen Y. Al-Kuwari N. Chen X. (2020). Association Between Parkinson’s Disease and Melanoma: putting the Pieces Together. *Front. Aging Neurosci.* 12:1–9. 10.3389/fnagi.2020.00060 32210791 PMC7076116

[B284] Zafar F. Valappil R. A. Kim S. Johansen K. K. Chang A. L. S. Tetrud J. W. (2018). Genetic fine-mapping of the Iowan SNCA gene triplication in a patient with Parkinson’s disease. *NPJ Parkins. Dis.* 4 1–7. 10.1038/s41531-018-0054-4 29928688 PMC6003950

[B285] Zamanian J. L. Xu L. Foo L. C. Nouri N. Zhou L. Giffard R. G. (2012). Genomic analysis of reactive astrogliosis. *J. Neurosci.* 32 6391–6410. 10.1523/JNEUROSCI.6221-11.2012 22553043 PMC3480225

[B286] Zhang W. Dallas S. Zhang D. Guo J.-P. Pang H. Wilson B. (2007). Microglial PHOX and Mac-1 are Essential to the Enhanced Dopaminergic Neurodegeneration Elicited by A30P and A53T Mutant Alpha-Synuclein. *Glia* 55 1178–1188. 10.1002/glia.20532 17600340

[B287] Zhang W. Wang T. Pei Z. Miller D. S. Wu X. Block M. L. (2005). Aggregated α−synuclein activates microglia: a process leading to disease progression in Parkinson’s disease. *FASEB J.* 19 533–542. 10.1096/fj.04-2751com 15791003

[B288] Zhang Y. Chen K. Sloan S. A. Bennett M. L. Scholze A. R. O’Keeffe S. (2014). An RNA-sequencing transcriptome and splicing database of glia, neurons, and vascular cells of the cerebral cortex. *J. Neurosci.* 34 11929–11947. 10.1523/JNEUROSCI.1860-14.2014 25186741 PMC4152602

[B289] Zhang Y. Sloan S. A. Clarke L. E. Caneda C. Plaza C. A. Blumenthal P. D. (2016). Purification and characterization of progenitor and mature human astrocytes reveals transcriptional and functional differences with mouse. *Neuron* 89 37–53. 10.1016/j.neuron.2015.11.013 26687838 PMC4707064

[B290] Zhu Y. Doornebal E. J. Pirtskhalava T. Giorgadze N. Wentworth M. Fuhrmann-Stroissnigg H. (2017). New agents that target senescent cells the flavone, fisetin, and the BCL-X-L inhibitors, A1331852 and A1155463. *Aging* 9 955–963. 10.18632/aging.101202 28273655 PMC5391241

